# Repeated evolution on oceanic islands: comparative genomics reveals species-specific processes in birds

**DOI:** 10.1186/s12862-024-02320-4

**Published:** 2024-11-08

**Authors:** María Recuerda, Julio César Hernández Montoya, Guillermo Blanco, Borja Milá

**Affiliations:** 1 Museo Nacional de Ciencias Naturales (MNCN), Consejo Superior de Investigaciones Científicas (CSIC), Calle José Gutiérrez Abascal 2, Madrid, 28006 Spain; 2 Cornell Laboratory of Ornithology, Cornell University, Ithaca, NY USA; 3 Grupo de Ecología y Conservación de Islas, A. C., Ensenada, Baja California México

**Keywords:** Comparative genomics, Island rule, Parallel evolution, Speciation

## Abstract

Understanding the interplay between genetic drift, natural selection, gene flow, and demographic history in driving phenotypic and genomic differentiation of insular populations can help us gain insight into the speciation process. Comparing patterns across different insular taxa subjected to similar selective pressures upon colonizing oceanic islands provides the opportunity to study repeated evolution and identify shared patterns in their genomic landscapes of differentiation. We selected four species of passerine birds (Common Chaffinch *Fringilla coelebs/canariensis*, Red-billed Chough *Pyrrhocorax pyrrhocorax*, House Finch * Haemorhous mexicanus* and Dark-eyed/island Junco *Junco hyemalis/insularis)* that have both mainland and insular populations. Changes in body size between island and mainland populations were consistent with the island rule. For each species, we sequenced whole genomes from mainland and insular individuals to infer their demographic history, characterize their genomic differentiation, and identify the factors shaping them. We estimated the relative (*F*_st_) and absolute (*d*_*xy*_) differentiation, nucleotide diversity (π), Tajima’s D, gene density and recombination rate. We also searched for selective sweeps and chromosomal inversions along the genome. All species shared a marked reduction in effective population size (N_e_) upon island colonization. We found diverse patterns of differentiated genomic regions relative to the genome average in all four species, suggesting the role of selection in island-mainland differentiation, yet the lack of congruence in the location of these regions indicates that each species evolved differently in insular environments. Our results suggest that the genomic mechanisms involved in the divergence upon island colonization—such as chromosomal inversions, and historical factors like recurrent selection—differ in each species, despite the highly conserved structure of avian genomes and the similar selective factors involved. These differences are likely influenced by factors such as genetic drift, the polygenic nature of fitness traits and the action of case-specific selective pressures.

**Supplementary Information:**

The online version contains supplementary material available at 10.1186/s12862-024-02320-4.

## Introduction

The colonization of oceanic islands by mainland individuals has been a major engine of biological diversification, resulting in the evolution of thousands of new plant and animal species across the world [1–5]. These colonization events have also provided valuable research models to study processes like evolutionary divergence and local adaptation [6–8]. Upon colonization of oceanic islands, individuals across taxonomic groups have often been subjected to similar demographic and selective factors, like founder effects, population bottlenecks, strong selection for local adaptation, and reduced dispersal [9, 10], resulting in examples of repeated evolution (e.g., [11, 12]). The concept of repeated evolution involves the evolution of similar traits in response to similar environmental pressures, and it encompasses processes like parallel and convergent evolution. Parallel evolution occurs when the ancestral state prior to selection is similar in both populations or taxa, whereas convergent evolution occurs when the ancestral state is different [13].

The molecular basis of similar phenotypic traits across species could be entirely species-specific, or instead show evidence of repeated evolution among species. The degree of repeated evolution at the molecular level can range from sharing the same mutation on the same gene, to changes at different nucleotides within the same gene, to changes in different genes within the same pathway [14, 15]. The probability of repeated molecular evolution is determined by several factors, increasing when selective pressures are similar and genomic constraints such as demography and phylogenetic history are shared [16]. The genetic architecture of the phenotypic traits under selection is also important: single-locus traits have been often involved in repeated evolution (e.g., [17, 18]), yet for polygenic traits, which can be modified through multiple pathways, repeated molecular evolution becomes less likely [16, 19, 20] resulting instead in heterogeneous, species-specific patterns of genetic differentiation. Therefore, when dealing with polygenic traits, it is more likely to observe repeated phenotypic evolution achieved by modifying different loci that are involved in similar functions [21, 22]. In insular environments, the effect of genetic drift and founder effects that reduce genetic diversity randomly, likely limits the number of loci selection can act on [23]. This, added to the fact that a smaller effective population size reduces the efficacy of selection, results in insular populations generally having a reduced adaptive potential compared to their mainland counterparts, constraining the possibilities of observing parallel molecular evolution in insular environments [24].

Understanding the factors that generate heterogeneous patterns of differentiation across the genome is one of the main goals of population genomics [25–29]. When comparing differentiated populations, regions that are highly divergent relative to the genomic background are known as “islands of differentiation” [30, 31] and are usually detected as regions of high relative divergence (*F*_st_ [32]). Recent advances in sequencing technologies have allowed studying the genomic landscapes of variation, which show the distributional pattern of genomic variation across the entire genome [23, 31, 33, 34]. The main factors shaping differentiation patterns are drift and selection, but demographic history and genomic features such as gene content and recombination and mutation rate, also affect the distribution of the differentiated regions [27]. Early genome scans interpreted *F*_st_ peaks as signatures of strong selection surrounded by valleys homogenized by gene flow [35], where those *F*_st_ peaks were caused by marked differences in allele frequencies at locally adapted sites and the neutral loci linked to them [36, 37]. However, when considering patterns of absolute divergence (*d*_*xy*_) and within-population diversity (π) besides *F*_st_, new interpretations of how these islands of differentiation originate have been put forward. *F*_st_ peaks could also appear when population diversity is low in either of the populations compared, while *d*_*xy*_ is less affected by this pattern. Several processes such as positive and/or background selection can reduce within population nucleotide diversity and generate “islands” of relative divergence, while absolute divergence remains unchanged [25, 38, 39]. Four theoretical models have been proposed to explain the underlying cause of islands of differentiation [39, 40] and to identify each one it is crucial to understand the relationship between *F*_st_, *d*_*xy*_ and π [25, 39–41]. Two of those models account for speciation in the presence of gene flow (“Divergence-with-gene-flow” and “Sweep-before‐differentiation”) and the other two involve allopatric speciation (“Selection in allopatry” and “Recurrent selection”) [40]. In the island colonization scenario, both speciation in the presence of gene flow and speciation in allopatry could potentially occur, yet in oceanic islands allopatric models are more likely. Thus, to correctly interpret the genomic landscapes of differentiation it is important to understand the demographic and evolutionary history of the target taxa [27]. Moreover, variations in effective population size (N_e_) can produce different genomic signatures. For instance, marked reductions in N_e_ such as those caused by population bottlenecks at founder events, can modify levels of background selection and therefore affect the baseline for the detection of outlier loci [24, 42].

Covariation of genomic patterns of differentiation among different avian species has been shown across broad evolutionary timescales [43–46] and the coincident location of differentiation peaks has been of special interest to understand the process of repeated molecular evolution where similar loci evolve independently in several species [47]. Bird genomes show high synteny [48], a relatively stable number of chromosomes [49], similar recombination landscapes [50, 51], and across species microchromosomes show higher density in gene content than macrochromosomes [50, 52]. The similarity in genomic landscapes of differentiation across closely related and recently diverged avian taxa could be attributed to a combination of factors. These include the non-random distribution of gene content across the genome and the coincidence of low recombination areas, along with linked selection, which may lead to the clustering of genes [44, 49]. Previous studies have demonstrated that the recombination landscape in birds can remain consistent across species over long evolutionary time periods [50].

To better understand the genomic underpinnings of phenotypic evolution, it is crucial to consider the role of chromosomal rearrangements, such as inversions. Inversions can keep sets of adaptive alleles together in strong linkage disequilibrium, promoting the maintenance of locally adapted genomic regions. Inversions can in some instances facilitate and accelerate the parallel adaptive process by making effective selection stronger [53]. The importance of inversions in repeated evolution has been demonstrated in various taxa (e.g., [54, 55]), and their role in avian evolution is receiving increased attention [56, 57], yet their role in the context of island colonization remains underexplored (but see [58]).

Repeated phenotypic changes on islands are often driven by similar selective pressures related to their unique features compared to mainland environments, such as simplified ecosystems, reduced trophic resources, the availability of new ecological niches, decreased predation (which can lead to increased intraspecific competition), and reduced interspecific competition [7, 59]. These insular selective pressures typically result in predictable changes, collectively known as the island syndrome [60, 61]. This syndrome includes modifications in body size [62], often attributed to the absence of predators and shifts in competition, as well as dietary changes to adapt to new trophic resources, leading to behavioral [63, 64], morphological [65, 66], and physiological adaptations [67, 68]. The consistent patterns of phenotypic evolution observed in insular populations across various taxonomic groups have given rise to general biogeographic rules, such as Foster’s rule, also known as the “island rule,” which posits that small animals tend to become larger and large animals tend to become smaller on islands [62, 69, 70].

These patterns suggest the potential for repeated evolutionary processes across species, providing an opportunity to investigate whether the selective mechanisms during island colonization are shared among species and whether selection targets the same or different genomic loci. However, newly established island populations often face significant genetic challenges due to founder effects and genetic drift, which can reduce their adaptive potential [24]. Small founding populations and random chance often lead to a substantial reduction in the genetic pool of colonizers compared to the source population [71]. The extent and persistence of this reduced diversity are influenced by several factors: the size of the founding population, the level of isolation, the number of colonization events, and the time elapsed since the initial migration, which allows for potential renewal of diversity through mutations and gene flow [72, 73]. Additionally, small effective population sizes, successive founder events, and frequent bottlenecks can further magnify the effects of genetic drift, leading to the rapid loss of genetic diversity [20, 74]. Understanding the genomic underpinnings of divergence in oceanic islands is complex due to the concurrent occurrence of multiple genomic processes. Nevertheless, recent advances in high-throughput DNA sequencing are enabling more studies to address this issue (reviewed in [15]). Both selection and drift play roles in influencing phenotypic changes in island populations, but patterns of repeated phenotypic change are more likely to be driven by selection rather than by random drift [16, 75].

Here we use a comparative approach to examine patterns of genome-wide differentiation in avian species that have colonized oceanic islands, with the goal of exploring if similar patterns of demographic history, time of divergence, and the effects of drift and directional selection in driving divergence could result in repeated molecular evolution upon island colonization. We selected four passerine species that have mainland populations and have also colonized oceanic islands; two species from mainland Europe that have colonized the island of La Palma in the Canary Islands, Atlantic Ocean, the Common Chaffinch (*Fringilla coelebs/canariensis*) and the Red-billed Chough (*Pyrrhocorax pyrrhocorax*), and two species from North America that have colonized Guadalupe Island on the Pacific Ocean, the House Finch (*Haemorhous mexicanus*) and the Dark-eyed Junco (*Junco hyemalis/insularis*). Given the considerable distance separating insular and mainland populations, and the fact that they are already recognized as separate subspecies or species, we can assume that the insular and mainland populations of these species are mainly allopatric with very restricted gene flow. The Red-billed Chough and the House Finch have diverged from mainland populations within the last 100,000 years, whereas the Common Chaffinch and the Junco have been separated from their mainland relatives for over 500,000 years [76–78]. Divergence times were estimated using mitochondrial DNA for all species, plus microsatellites for the Red-billed Chough and SNPs for the Common Chaffinch. Given that all four species have colonized oceanic islands and have been subjected to potentially similar selective pressures, we first analyzed if the differences in morphology between insular and mainland counterparts affected the same traits across species, as specific changes in morphological traits are expected upon colonization of the new insular environment [4, 10].

We also asked if the genomic landscapes of differentiation are similar among species with different divergence times between insular and mainland counterparts. We performed whole-genome resequencing of 9–12 individuals per treatment (i.e., island/mainland) per species to determine whether the four species showed similar patterns of differentiation in their genomic landscapes, and whether these patterns have been shaped by similar processes. We studied the demographic history and performed genomic scans of *F*_st_, *d*_*xy*_, π, Tajima’s D, recombination rate, gene content and selective sweeps. We also scanned the genomes looking for putative chromosomal inversions, which have been shown to underlie repeated evolution in birds [79]. We detected regions under selection among insular and mainland counterparts as *F*_st_ outliers, and identified shared candidate genes among the four species. Comparing the genomic signatures of island colonization in four different species that have been exposed to similar selective pressures and that differ in colonization/divergence time (which can be considered as a proxy for different stages along the speciation continuum due to reproductive isolation in allopatry), can provide useful understanding for the mechanisms shaping the genomic landscapes through the divergence process over time.

## Methods

### Study area and fieldwork

**Fig. 1:**
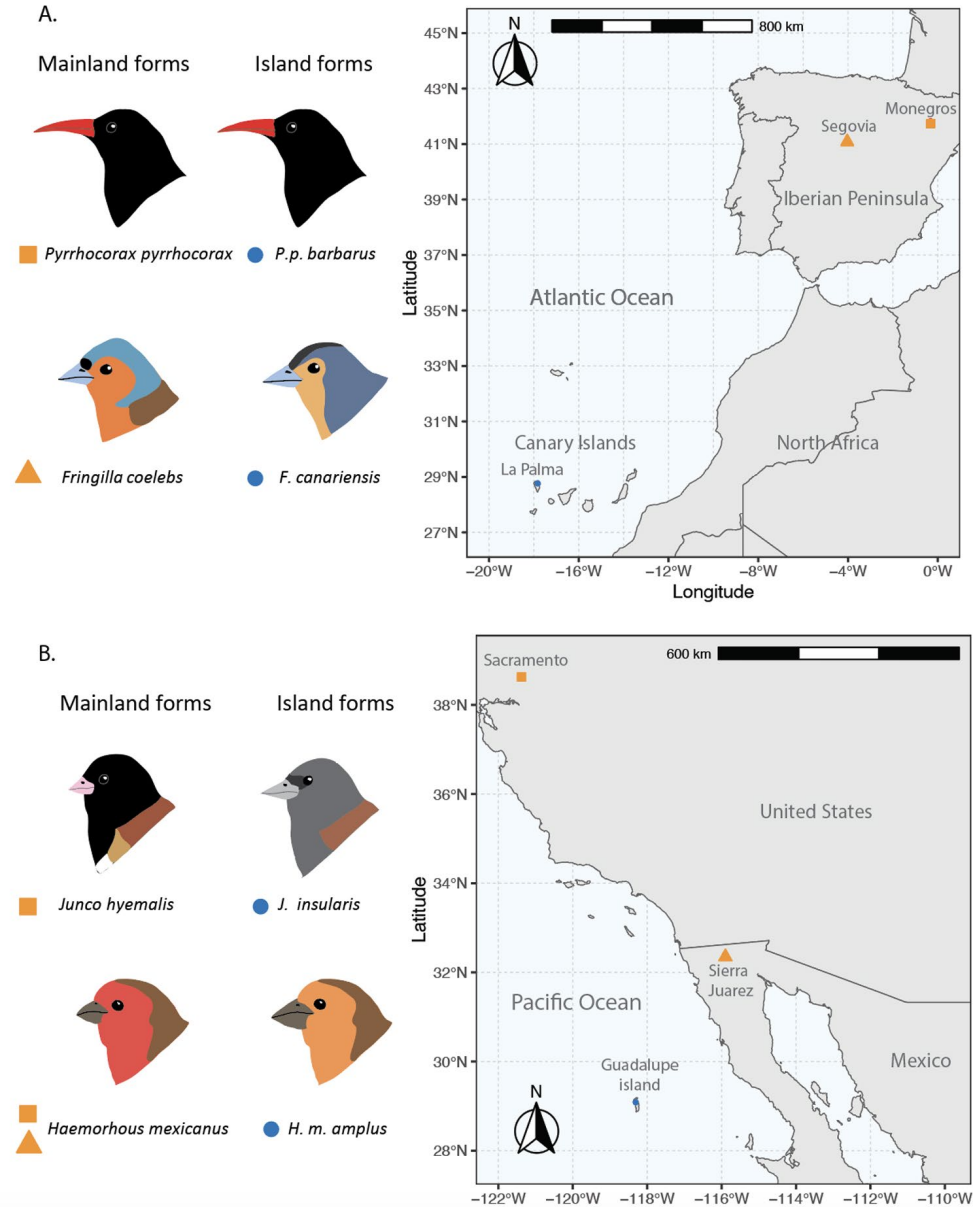
Target taxa for comparative analysis. (**A**) Species that have colonized La Palma in the Atlantic Ocean: Red-billed Chough and Common Chaffinch. (**B**) Species that have colonized Guadalupe island in the Pacific Ocean: Dark-eyed Junco and House Finch. Nomenclature according to Clements et al., [178]. The shape and colors of markers next to the names correspond to the sampling locations on the map, with orange rectangles and triangles for the continental species and blue circles for the insular taxa

We sampled mainland populations of the Common Chaffinch (Fringillidae: *Fringilla coelebs*) and the Red-billed Chough (Corvidae: *Pyrrhocorax pyrrhocorax*) in the Iberian Peninsula at Segovia and Los Monegros, respectively (see [77, 78]). The insular populations from both species were sampled in La Palma, the most north-western island of the Canary Islands archipelago (Fig. 1A, Table S1). The Common Chaffinch lineage in the Canary Islands has recently been raised to species status [80], and we use its current name, Canary Islands Chaffinch (*Fringilla canariensis*). The mainland populations of the House Finch (Fringillidae: *Haemorhous mexicanus*) and Dark-eyed Junco (Passerellidae: *Junco hyemalis oreganus*) were sampled in California, and two House Finch individuals were sampled in Sierra Juarez (Baja California, Mexico). Insular populations for both species were sampled in Guadalupe Island, Mexico, in the Pacific Ocean (Fig. 1B, Table S1). The Junco on Guadalupe Island, until recently a subspecies of *J. hyemalis*, has been raised to species status, and we use its current name, Island Junco (*Junco insularis*).

All individuals were captured in the field using mist nets and mesh traps in the case of Red-billed Choughs. All individuals were marked with uniquely numbered aluminum bands, sexed, aged and measured in the field. A blood sample was obtained by venipuncture of the sub-brachial vein and stored in absolute ethanol at -20 °C in the laboratory for DNA extraction. After processing, birds were released unharmed at the site of capture. As the red-billed chough was the only non-dimorphic species, we determined its sex by the amplification of the Chd1 gene following Griffiths et al. [81].

### Morphological data and analysis

Morphological traits in adult males from mainland and insular populations were measured across all species. For the Common Chaffinch, the Junco and the House Finch a wing ruler was used to measure unflattened wing length to the nearest 0.5 mm, and dial callipers of 0.1-mm precision were used to measure tail length, tarsus length, bill culmen, total bill length, bill width and bill depth, following Milá et al. [82]. All measurements were taken by a single observer (BM). For the Red-billed Chough, the same traits were measured by a single observer (GB) following standard methods described previously [83]. We compared the morphological traits of adult males from mainland and insular populations for all species using principal components analysis (PCA) of all variables and univariate analysis of variance (ANOVA) to compare the means among treatments for each species. The PCA including all morphological variables was computed using the *prcomp* function in *stats* v. 3.6.2 R package.

### Genome resequencing

Genomic DNA was extracted with a QIAGEN Blood and Tissue kit following the manufacturer’s protocol. Whole genome resequencing at 18x coverage of 24 individuals per species (12 per treatment, but only 9 for the mainland Common Chaffinch) was conducted on a SE50 Illumina™ platform at Novogene™. Reads were trimmed with *Trim Galore* [84] with default settings and a minimum length of 40 and then mapped to their respective reference genomes using BWA (Burrows-Wheeler Aligner [85]. For the Common Chaffinch and the House Finch we used the Common Chaffinch reference genome (GCA_015532645.2 [86]); for the Junco we used the *Junco hyemalis* reference genome (GCA_003829775.1 [87]); and for the Red-billed Chough we used the *Corvus moneduloides* reference genome (GCA_009650955.1, bCorMon1.pri). The Maximum likelihood genetic distance between the House Finch and the Common Chaffinch is 0.12 (16.5 Mya) [88] and between the Red-billed Chough and the *Corvus moneduloides* is around 5.9-8 Mya [89]. SNPs were called using BCFTOOLS v.1.3.1 [90] including invariant sites. Filtering was performed with VCFTOOLS v. 0.1.15 [91] separately for variant and invariant sites, using the following criteria for variant sites: (i) indels and sites with more than two alleles were removed; (ii) a number of reads per site between 10 and 40; (iii) a minimal genotype quality of 30; (iv) a minor allele count of 2 (--mac 2); and (v) 25% maximum missing data and for invariant sites a minimal genotype quality of 30. Variant and invariant sites were then merged using BCFTOOLS concat. The reference genomes from all four species were aligned to the zebra finch genome (*Taeniopygia guttata*, bTaeGut2.pat.W.v2) using nucmer from the MUMmer package (v.4.0, ‘-b 400’ and filtering with ‘delta-filter − 1’); [92] and chromosomes were numbered accordingly (see Table S2, Fig. S1).

### Inference of demographic history

The change in effective population size (N_e_) across time for each species was estimated using Pairwise Sequentially Markovian Coalescent (PSMC) analysis [93]. The PSMC model infers demographic history based on genome-wide heterozygous sequence data. We used SAMTOOLS [94] to obtain diploid consensus sequences from BAM files generated with BWA-mem [85]. Sites with sequencing depth lower than 10 and higher than 35 were removed. Because sex chromosomes can show different rates and patterns of evolution than autosomes (reviewed by [39, 95]), we focused our comparisons of differentiation statistics on autosomes only. We converted the diploid consensus sequence to PSMC input files (psmcfa) using the tool fq2psmcfa included in the PSMC software. Then, the program PSMC was used to infer the population history with the options ‘-N25 –t5 –r1 –p “4 + 30*2 + 4 + 6 + 10’, except for the mainland Common Chaffinch, and for both populations of the House Finch, where the upper time limit was set to 1 (-t1) to achieve convergence. We performed 100 bootstraps for one genome per species and treatment. The atomic time interval was set following Nadachowska-Brzyska et al., [96]. We used a mutation rate of 4.6 × 10^− 9^ mutations/site/generation [97], which has been used in other avian systems for PSMC analysis (e.g., [66, 98–100]). Generation time was set to two years for all species [101–103]. We also performed a test setting the generation time to three years for the Red-billed Chough and to one year for the Common Chaffinch, the Junco and the House Finch and to explore how this variability would affect divergence time estimates (Fig. S3).

### Inference of recombination rate

To determine the effect of recombination rate on the genomic landscapes of differentiation, we estimated recombination rates across the genome for insular and continental populations for the four species using LDhat software [104]. First, we created a modified likelihood lookup table based on the LDhat precomputed tables using a sample size of 12 per treatment (9 for the continental Common Chaffinch) and a population mutation rate parameter estimate of 0.001. Then vcf files were split into chunks of 10,000 SNPs and converted to ldhat format using VCFTOOLS v. 0.1.15 [91]. The input files generated were used in LDhat “interval” to estimate the effective recombination rate by implementing a Bayesian MCMC sampling algorithm with five million iterations, sampling every 5,000 steps and a block penalty of 10. Finally, the results were summarized using the LDhat module “stat”, discarding 20% of the samples as burn-in.

### Genome scans and detection of selective sweeps

In order to detect genomic signatures of selection among the island and mainland counterparts from the four different species, we estimated two different statistics, the fixation index (*F*_st_ [32]), and the cross-population extended haplotype homozygosity (XP-EHH) [105]. First, *F*_st_, *d*_*xy*_ and π using were calculated in non-overlapping windows of 10Kb using pixy v. 2 [106]. Pixy takes into account the invariant sites for π and *d*_*xy*_ calculations, thus overcoming the problem of most programs that use VCF files to calculate those statistics but do not distinguish among invariant and missing sites, resulting in deflated estimates [106]. We also computed Tajima’s D [91, 107] in non-overlapping 10-Kb windows with VCFTOOLS [91, 108]. To detect outliers for all variables, first the averaged values of each variable were transformed to Z-scores using the *scale* command in R and then the p-value was calculated with the *pnorm* function to correct for multiple testing setting the false discovery rate (FDR) to 0.05 [108].

To detect selective sweeps, we computed the cross-population extended haplotype homozygosity (XP-EHH) [105], using the R package *rehh* v.3.2.2 [109]. First, we phased the vcf files containing only the variant sites in 50-Kb windows using Shapeit v2.r904 [110]. The XP-EHH is based on the comparison of haplotype lengths between populations and has most detection power when the selected haplotype is near fixation in one population and still polymorphic in the other. The genomic regions showing a –log10(p-value) ≥ 3 (i.e., *p* ≤ 0.001) were considered to be under selection. Then, we looked for overlapping regions between the *F*_st_ and the XP-EHH outliers. We generated Manhattan plots for all the statistics using the R package *qqman* [111] in R. All R analyses were performed in v. 3.6 [112].

### Detecting putative chromosomal inversions

We examined how patterns of population structure varied along the genome to detect potential chromosomal inversions using the R package *lostruct* v.0.0.4 [113]. This method can help identify putative chromosomal inversions; however, it does not provide definitive evidence of their existence. SNP data for each species including only variant sites was converted to BCF format using BCFTOOLS version 1.9 [94]. We implemented the script provided by Huang et al., [114] dividing the genome into 1,000-SNP non-overlapping windows and applying a principal components analysis (PCA) to each window. Euclidian distances between the two first principal components (PCs) between windows were calculated and mapped using multidimensional scaling (MDS) into a 40-dimensional space to see the similarity of the relatedness patterns between windows. To identify genomic regions with extreme MDS values, windows with absolute values greater than 4 SD over the mean across all windows were selected for each MDS coordinate. We performed 1,000 permutations of windows over chromosomes to test if outlier regions were randomly distributed across chromosomes. The putative inversion coordinates were the start position of the first outlier window and the end position of the last outlier window. The script included additional analyses to check if the MDS outliers were detecting inversions or instead other processes such as linked selection. First, a PCA was performed using the SNPs from each putative inversion with *SNPRelate* [115]. Inversions in the PCA would split the samples into three different groups (i.e., the two orientations and the heterozygotes in an intermediate cluster). The R function *kmeans* with the Hartigan & Wong [116] method was used to identify the composition of groups of genotypes by performing clustering on the first PC, setting the initial cluster centers as the maximum, minimum and middle of the PC score range. Then, another test was performed averaging the individual heterozygosity per group detected by the k-means clustering. Inversions would show a pattern of higher heterozygosity of the central group relative to the other two groups. In addition, LD heatmaps of the putative inversion regions were constructed using the LDBlockShow v1.40 [117]. Finally, only MDS outlier regions that clustered into three groups in the PCA and showed higher heterozygosity in the middle group and high LD within the estimated regions were considered as putative inversions.

### Candidate genes and GO-term enrichment analysis

We extracted the candidate genes of the genomic regions detected to be under selection by both methods separately (*F*_st_ and XP-EHH outliers) using bedtools intersect and the annotation of their respective reference genomes. We checked their functions in *genecards* [118]. We obtained the GO terms using the Zebra Finch dataset in *biomaRt* in R. We then performed a Gene ontology (GO) enrichment analysis for each set of outliers in the category “biological function” using the *TopGO* v.2.50.0 R package [119]. To estimate the statistical significance, we used the Fisher exact test implementing the *weight01* method. As recommended by the *TopGO* authors, we did not implement corrections for multiple testing and presented raw p-values for the top-10 GO terms related to biological processes.

## Results

### Morphological differences

**Fig. 2:**
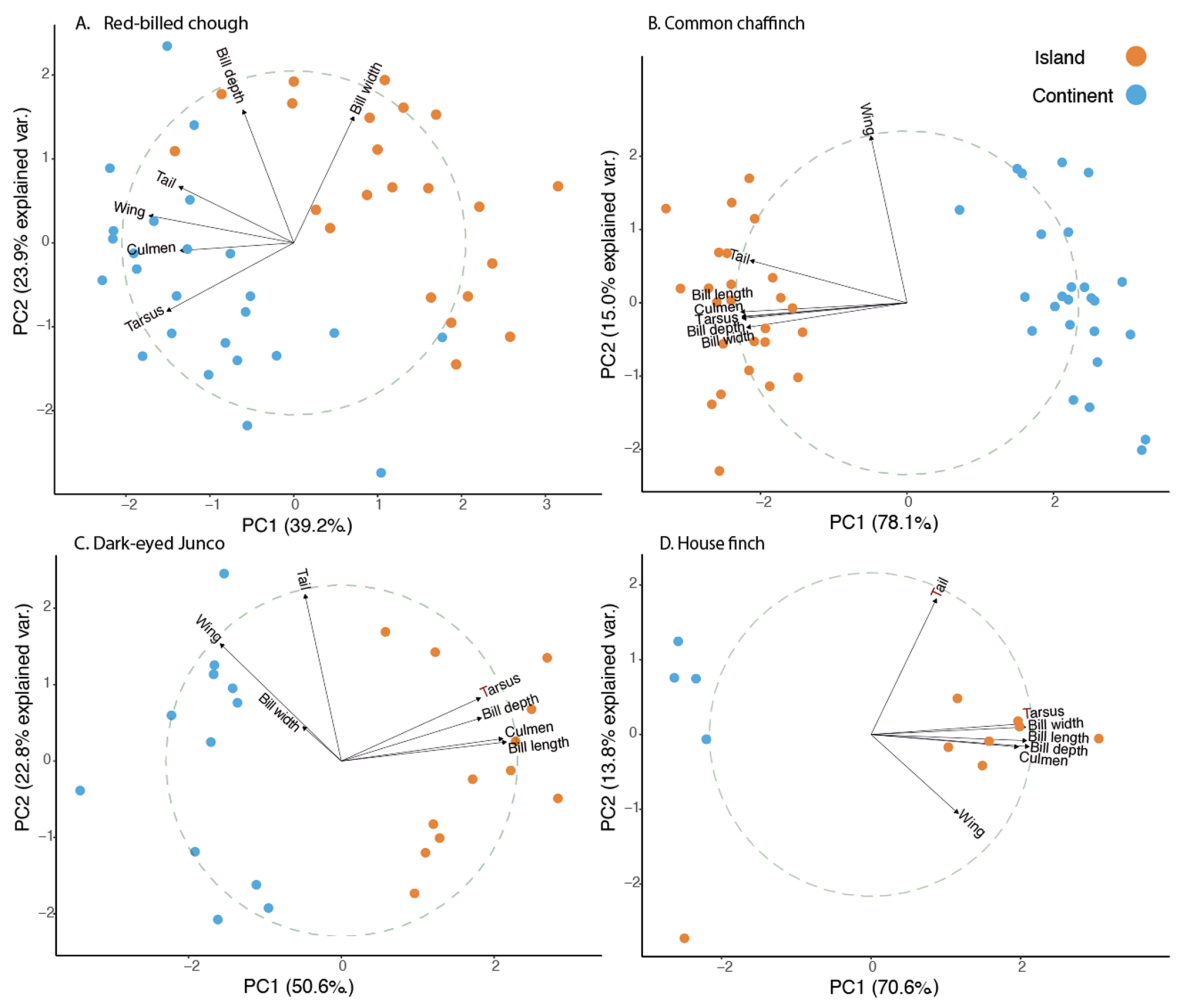
Principal Components Analysis (PCA) with morphological data per species **A**) Red-billed Chough, **B**) Common/Canary Islands Chaffinch, **C**) Dark-eyed/island Junco, **D**) House Finch. The variables included are wing, tail and tarsus length and bill culmen, bill depth, bill width and bill length (the latter is not included for the Red-billed Chough). The correlation circle with radius 1 show the loadings of each variable that are represented by the arrows. Red and blue markers correspond to insular and mainland individuals, respectively

The morphological analysis revealed marked differences in most traits between insular and continental populations for all species. The threshold to consider small and large bird species in the context of the island rule has been shown to be around 60 g [62]. The smaller species (Common Chaffinch, Junco, and House Finch), shared a pattern of significantly larger values for most traits in the insular populations compared to mainland, except for the Junco wing length, which was longer in the continent (Table S3). We detected the opposite pattern in the larger sized Red-billed Chough, with significantly smaller values for most morphological traits in the insular populations, except for bill width which was smaller in the continent (Table S3). PC1 values showed marked separation of insular and mainland populations in all four species, explaining between 39 and 78% of the morphological variance (Fig. 2).

### Whole-genome resequencing

The total number of sites obtained in the variant calling was close to the length of the reference genomes. The number of variant sites (40–50 million) was similar for all species except for the Red-billed Chough, which was lower (~ 13 million), and the same pattern was maintained after filtering (Table S4). The lower number of variants of the Red-billed Chough is consistent with the lower ancestral effective population size compared to the other species [120].

### Inference of demographic history

**Fig. 3:**
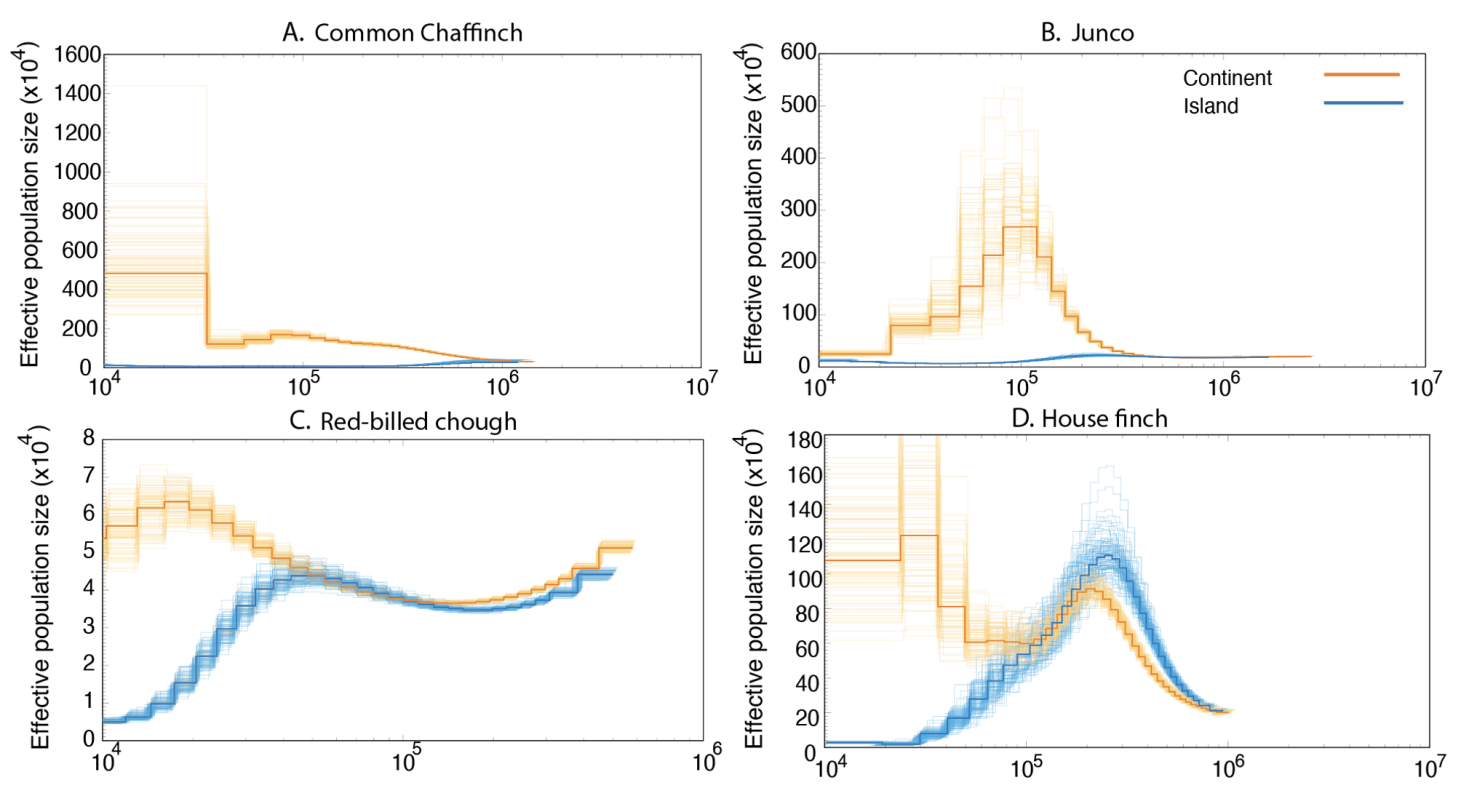
Demographic history of insular and mainland populations. The analysis was performed using Pairwise Sequentially Markovian Coalescent (PSMC). Demographic inference for one individual per treatment and species, with the orange and blue dark lines corresponding to the continental and insular populations, respectively. Shown are PSMC plots for **A**) Common Chaffinch, **B**) Junco, **C**) Red-billed Chough and **D**) House Finch. The lighter orange and blue lines represent 100 bootstrap replicates. The point where both lines depart from each other corresponds to the time of colonization, which is around 40,000 y for the Red-billed Chough, 900,000 y for the Common Chaffinch, 100,000 y for the House Finch and 400,000 y for the Dark-eyed Junco. The mutation rate used was of 4.6e-9 mutation/site/generation for all species, and the generation time used in all cases was two years. See Fig. S2 for bootstrapped versions of the individual PSMC plots. See Fig. S3 for PSMC plots with generation time of one year for the Common Chaffinch, Dark-eyed Junco and House Finch and three years for the Red-billed Chough

PSMC-based demographic inference revealed a consistent pattern for the four species, showing stable or growing effective population sizes for mainland populations and a sharp reduction in effective population size in insular populations following colonization. The island-mainland divergence time estimates obtained from the PSMC analysis are around 900,000 years for the Common Chaffinch, 100,000 years for the House Finch, 400,000 years for the Dark-eyed Junco, and 30,000 years for the Red-billed Chough (Fig. 3, S2). To account for the variability in generation time, we applied a generation time of three years for the chough and one year for the rest of the species, obtaining different split time estimates but still within the expected values: 70,000 years for the Red-billed Chough, 500,000 years for the Common Chaffinch, 200,000 years for the Dark-eyed Junco, and 80,000 years for the House Finch (Fig. S3). The continental population of the Red-billed Chough showed the smallest effective population size, and the smallest difference between the continental and insular populations among the study species.

### Inferring parallel molecular evolution from genome-wide scans

**Fig. 4:**
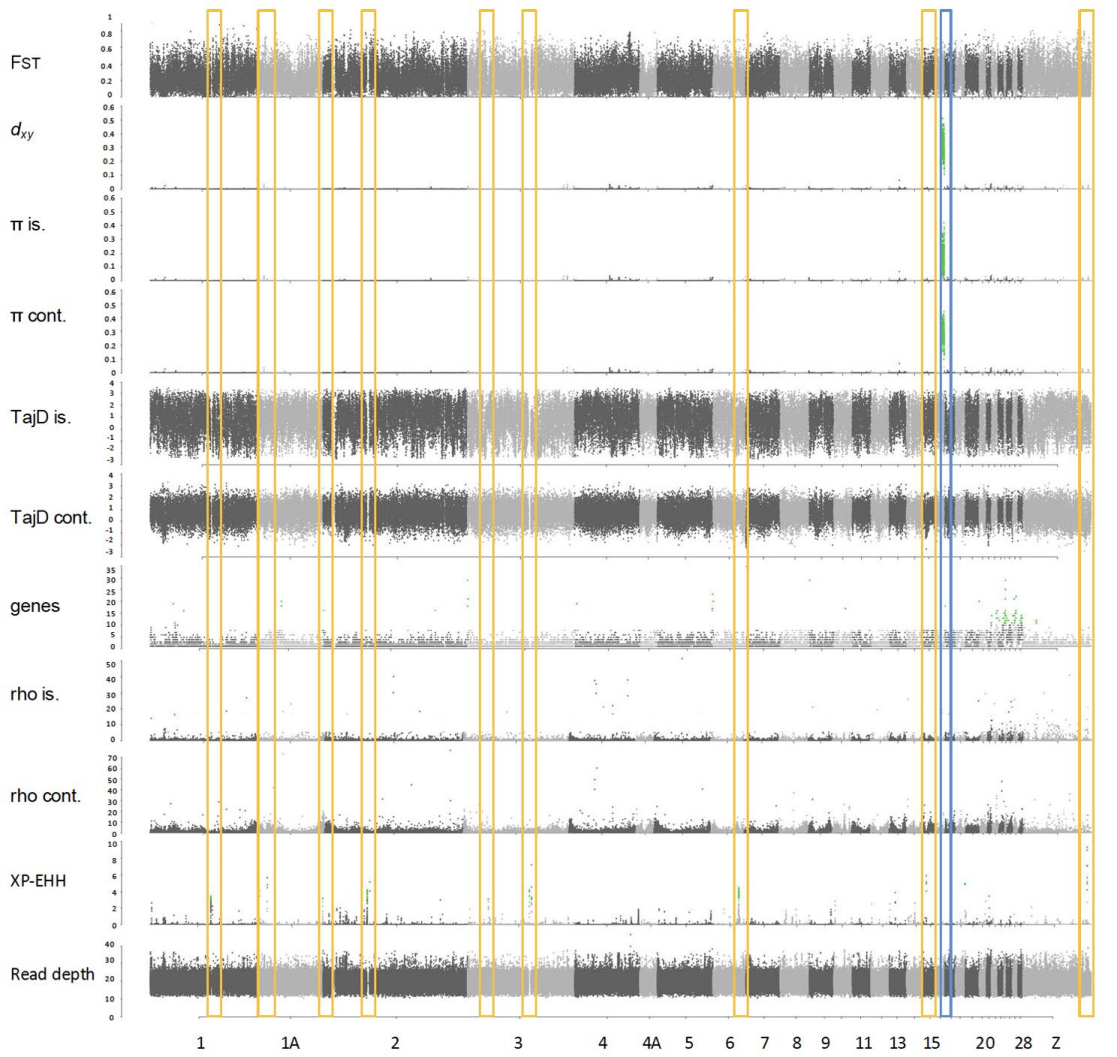
Genomic scans for several summary statistics for an island-mainland comparison in the Red-billed Chough (*Pyrrhocorax pyrrhocorax*). From top to bottom, fixation index (*F*_st_), genomic divergence (*d*_*xy*_), genetic diversity for insular and continental populations (π), Tajima’s D for insular and continental populations (TajD), number of genes, recombination rates for insular and mainland populations (rho), cross-populations extended haplotype homozygosity (XP-EHH) and read depth. Chromosome numbers correspond to the Zebra Finch genome (*Taeniopygia guttata*). Green dots represent outliers with the false discovery rate (FDR) set at 0.05 after applying the Benjamini and Hochberg correction, except for the XP-EHH, where the threshold is set at –log10 (*p*-value) ≥ 3. The yellow boxes highlight the XP-EHH peaks coincident with regions with no coverage. The blue box correspond to the region with high absolute divergence (*d*_*xy*_ ) and genetic diversity (π) in chromosome 17

**Fig. 5:**
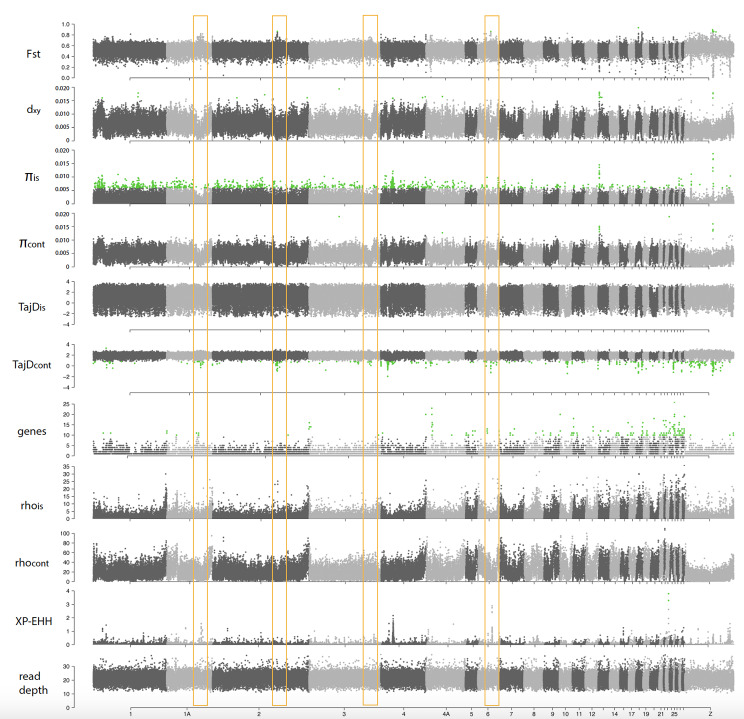
Genomic scans for several summary statistics for an island-mainland comparison in the Common Chaffinch (*Fringilla coelebs*). From top to bottom, fixation index (*F*_st_), genomic divergence (*d*_*xy*_), genetic diversity for insular and continental populations (π), Tajima’s D for insular and continental populations (TajD), number of genes, recombination rates for insular and mainland populations (rho), cross-populations extended haplotype homozygosity (XP-EHH) and read depth. Chromosome numbers correspond to the Zebra Finch genome (*Taeniopygia guttata*). Green dots represent outliers with the false discovery rate (FDR) set at 0.05 after applying the Benjamini and Hochberg correction, except for the XP-EHH, where the threshold is set at –log10 (*p*-value) ≥ 3. The yellow boxes highlight the signatures of recurrent selection (*F*_st_ peaks coincident with drops in *d*_*xy*_ and π). Some of them are also coincident with peaks in XP-EHH

**Fig. 6:**
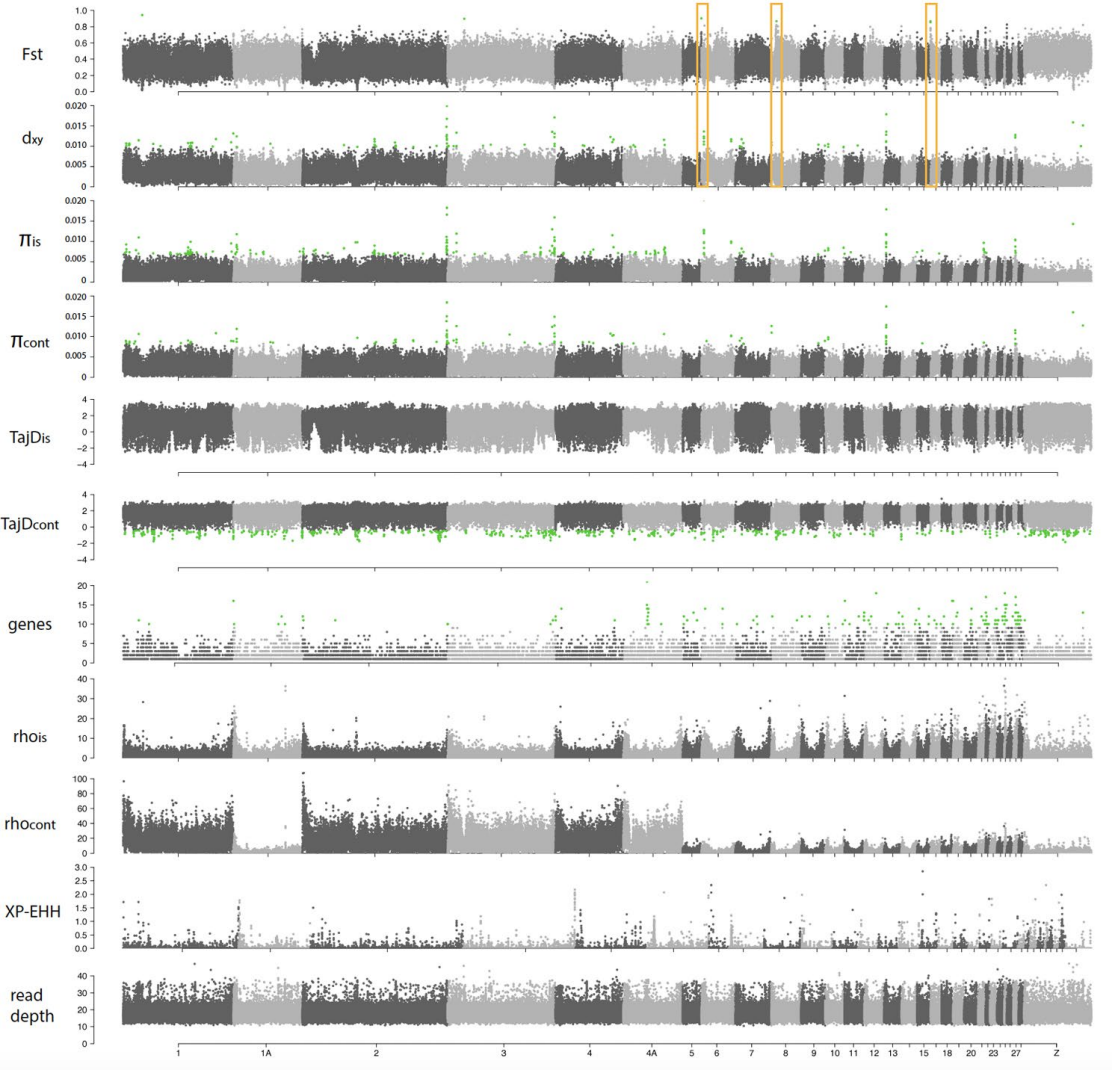
Genomic scans for several summary statistics for an island-mainland comparison in the Dark-eyed Junco (*Junco hyemalis*). From top to bottom, fixation index (*F*_st_), genomic divergence (*d*_*xy*_), genetic diversity for insular and continental populations (π), Tajima’s D for insular and continental populations (TajD), number of genes, recombination rates por insular and mainland populations (rho), cross-populations extended haplotype homozygosity (XP-EHH) and read depth. Chromosome numbers correspond to the Zebra Finch genome (*Taeniopygia guttata*). Green dots represent outliers with the false discovery rate (FDR) set at 0.05 after applying the Benjamini and Hochberg correction, except for the XP-EHH, where the threshold is set at –log10 (*p*-value) ≥ 3. The yellow boxes highlight the *F*_st_ peaks in the chromosome extremes

**Fig. 7:**
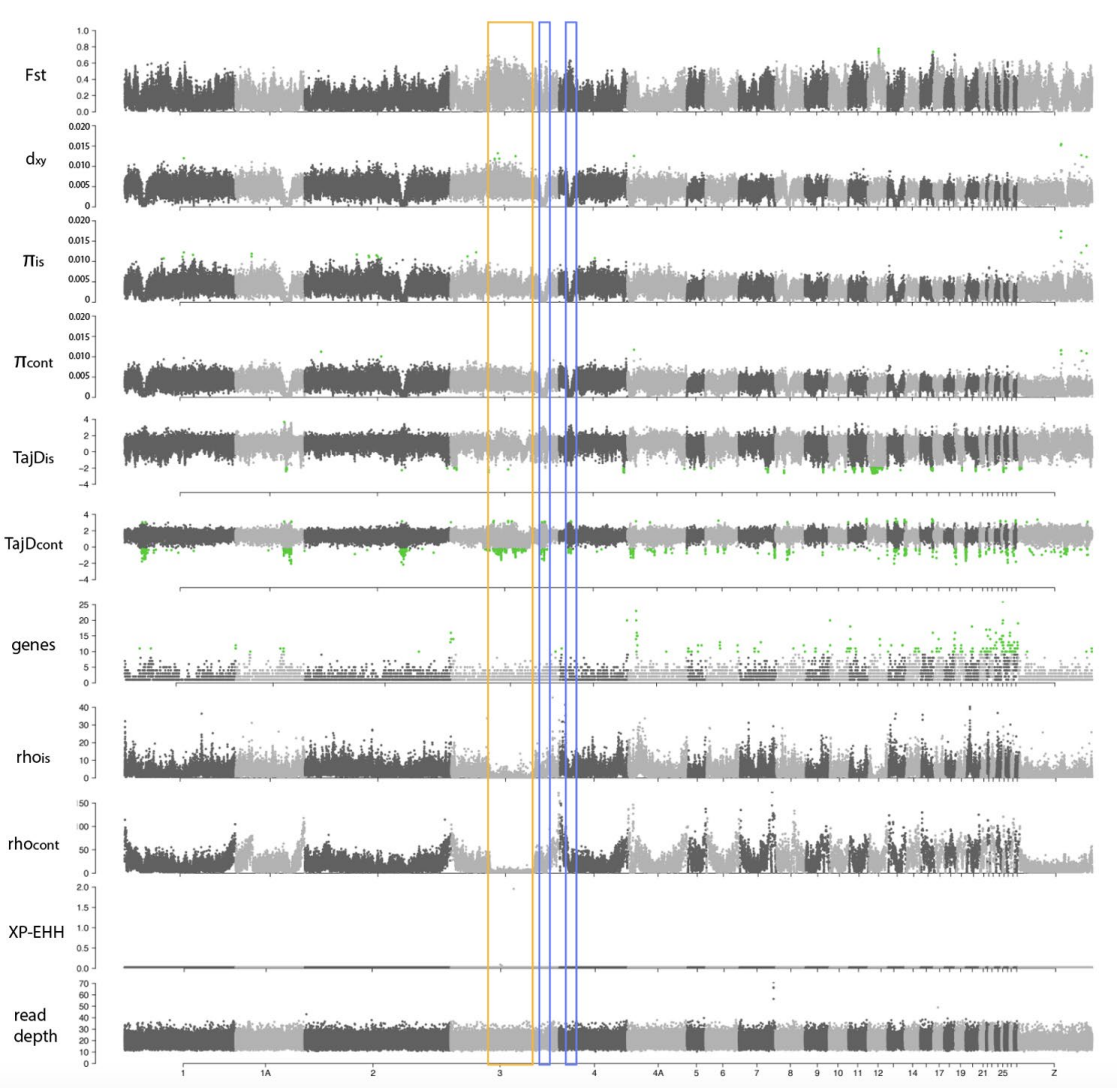
Genomic scans for several summary statistics for an island-mainland comparison in the House Finch (*Haemorhous mexicanus*). From top to bottom, fixation index (*F*_st_), genomic divergence (*d*_*xy*_), genetic diversity for insular and continental populations (π), Tajima’s D for insular and continental populations (TajD), number of genes, recombination rates por insular and mainland populations (rho), cross-populations extended haplotype homozygosity (XP-EHH) and read depth. Chromosome numbers correspond to the Zebra Finch genome (*Taeniopygia guttata*). Green dots represent outliers with the false discovery rate (FDR) set at 0.05 after applying the Benjamini and Hochberg correction, except for the XP-EHH, where the threshold is set at –log10 (*p*-value) ≥ 3. The yellow box highlights the putative inversion in chromosome 3 (*F*_st_ peak that coincides with a drop in the recombination rate). The blue boxes highlight the signatures of recurrent selection (*F*_st_ peak coincident with drops in *d*_*xy*_ and π)

Genome-wide scans of genetic differentiation showed high heterogeneity across the four target species. The *F*_st_ genomic landscapes varied strongly among species (Figs. 4, 5, 6 and 7). Mean *F*_st_ was higher in the Common Chaffinch, followed by the Dark-eyed Junco, as expected for relatively longer island-mainland divergence times. The Red-billed Chough showed a slightly higher mean *F*_st_ than the House Finch (Table 1). The Red-billed Chough’s absolute divergence and genetic diversity for insular and mainland populations were the lowest but comparable to the rest, mainly due to a specific region of about 3.4 Mb in chromosome 17 with exceptionally high *d*_*xy*_ and genetic diversity. When this region is excluded, both *d*_*xy*_ and π drop to an order of magnitude lower than those of the other species (Table 1). The continental Common Chaffinch population showed the highest diversity value (Table 1). All species showed consistently higher gene content and recombination rates at microchromosomes, and in general, recombination rates were higher at chromosome ends (Figs. 4, 5, 6 and 7).

**Table 1:** Divergence and diversity across the genome. Mean values, standard deviation (sd) and range of genomic summary statistics for the four species including the Red-billed Chough without the region with high absolute divergence and genetic diversity. The table includes sample sizes for the continental and insular populations (N_cont_ and N_is_), fixation index (*F*_**st**_), absolute genomic divergence (*d*_*xy*_), and genetic diversity (π) for the insular and the continental populations

Species	*N*_cont_	*N*_is_	F_st_ ± sd	range	d_xy_ ± sd	range	π_island_ ± sd	range	π_continent_ ± sd	range
Red-billed Chough	12	12	0.24 ± 0.13	[-0.062–0.89]	0.002 ± 0.02	[0–0.54]	0.001 ± 0.01	[0–0.42]	0.002 ± 0.02	[0–0.44]
Red-billed Chough_nopeakchr17	12	12	0.24 ± 0.13	[-0.062–0.89]	0.0009 ± 0.0009	[0–0.065]	0.0005 ± 0.0008	[0–0.066]	0.0008 ± 0.0009	[0–0.068]
House Finch	12	12	0.16 ± 0.10	[-0.06–0.69]	0.005 ± 0.002	[0–0.017]	0.004 ± 0.002	[0–0.018]	0.005 ± 0.002	[0–0.016]
Dark-eyed Junco	12	12	0.29 ± 0.08	[-0.060–0.77]	0.004 ± 0.002	[0–0.023]	0.002 ± 0.001	[0–0.023]	0.004 ± 0.002	[0–0.022]
Common Chaffinch	9	12	0.44 ± 0.05	[-0.033–0.93]	0.008 ± 0.003	[0–0.021]	0.002 ± 0.001	[0–0.020]	0.008 ± 0.003	[0–0.022]

The genomic landscape of the Red-billed Chough shows high levels of relative differentiation across the whole genome, with no clear outlier regions. Mean genetic diversity in both populations is generally very low throughout the genome, except for a highly differentiated region on chromosome 17. The XP-EHH analysis indicated evidence of selective sweeps, with a few distinct peaks along the genome (Fig. 4). However, most of these peaks are adjacent to regions of the genome with no coverage, probably corresponding to centromeric/high repeat content regions; and are therefore likely artifacts (Fig. S4).

The Common Chaffinch genome landscape is characterized by several *F*_st_ peaks that coincide with valleys in *d*_*xy*_ and π, and negative peaks in Tajima’s D mainly in the continent (i.e., peaks in chromosomes 1,1 A, 2, 3, 4, 4 A, 6, Fig. 5). This pattern is consistent with the model of recurrent selection, which states that selection in the ancestral population prior to the mainland-island split generates a pattern of low *d*_*xy*_. Subsequent selection in those regions after divergence further reduces genetic diversity, generating *F*_st_ peaks. XP-EHH revealed peaks suggesting selective sweeps mostly concentrated in the microchromosomes and the Z chromosome and few of them coincided with the regions of recurrent selection.

The Dark-eyed Junco genomic landscape is highly differentiated across the entire genome, and there are few outlier genomic regions, which often coincide with chromosomal ends (Fig. 6). The XP-EHH scans did not detect significant selective sweeps across the genome, but there is again a pattern of peaks in the chromosomal ends.

The House Finch genomic landscape is characterized by a large, highly differentiated region in the middle of chromosome 3, representing 47 million base pairs, suggesting a large chromosomal inversion. It coincides with significant positive and negative peaks of Tajima’s D in the continental population and a region of low recombination, while *d*_*xy*_ and π show regular values (Fig. 7). At the end of the same chromosome and at the beginning of chromosome 4, there are two *F*_st_ peaks that coincide with a valley in *d*_*xy*_ and π, and a peak in Tajima’s D. This pattern is consistent with the recurrent selection model. In chromosomes 1, 1 A and 2 there is a similar pattern but, in these cases, there is no *F*_st_ peak. The microchromosomes show high relative differentiation along with high recombination rates and enriched gene content. The XP-EHH scan showed a relatively flat landscape with no evidence for significant selective sweeps.

### Detecting putative chromosomal inversions

After combining all possible evidence, the analysis to detect inversions revealed that the Red-billed Chough genome has no putative inversions. The Dark-eyed Junco genome showed five possible inverted regions distributed in chromosomes 1, 3 and 5 (Table S5, Fig. S5) and the one in chromosome 1 coincided with an *F*_st_ outlier region. The Common Chaffinch genome showed two possible inversions in chromosomes 10 and 20, and the two of them coincided with *F*_st_ outlier regions (Table S5, Fig. S6). The House Finch genome revealed four putative inversions, with one particularly large in chromosome 3. (Table S5, Fig. S7). Only two putative inversions coincide with *F*_st_ outlier regions, the large inversion in chromosome 3 and a 21 Mb inversion in chromosome Z.

### Detection of candidate genes and GO-term enrichment analysis

Sharing of candidate genes among species was limited (Table S10). There were only five genes putatively under selection that were shared between different species: the *morc2* gene was shared between the House Finch and the Dark-eyed Junco; and the clic3, paxx, tmem141 and entp2 genes were shared between the House Finch and the Red-billed Chough *d*_*xy*_ outliers. The *morc2* gene is associated with Marie-Tooth disease, axonal, type 2z (CMT2Z) and developmental delay, impaired growth, dysmorphic facies, and axonal neuropathy (DIGFAN) diseases in humans. CMT2Z is characterized by distal lower limb muscle weakness and sensory impairment [121] and DIFGAN by impaired motor and intellectual development, poor overall growth, usually short body height and microcephaly and subtly dysmorphic facial features in humans [122, 123]. The clic3 gene has been associated with bone formation in humans [124]. The paxx and tmem141 genes are involved in neural development [125, 126]. The entp2 gene has been found to be related to taste transduction in core landbirds [127].

The number of genes included in the *F*_st_ outlier regions ranged from 2 to 84 and in all cases except for the Red-billed Chough were higher than the number of genes in the XP-EHH outlier regions (Table 2). Only in the Common Chaffinch there was an overlap of 1 gene between both methods (Table S10). In the Red-billed Chough, due to the high relative differentiation across the genome, the absence of clear *F*_st_ peaks, and the potential artifacts in selective sweep detection caused by regions with no coverage, focusing on the peak of absolute genomic differentiation could be a more effective approach to identify candidate regions in this species. For the *d*_*xy*_ outliers, the top-10 GO terms are related to the regulation of signaling pathways, ion transport, and protein modifications (Table S6). In the Common Chaffinch, among the top-10 GO terms we found several involved in transmembrane transport, protein modification processes and organization of nuclear and cellular components (Table S7). In the Dark-eyed Junco, the top-10 GO terms revealed several involved in the regulation of cellular and molecular localization including two terms related to the centrosome and related to nervous system development and function (Table S8). Finally, in the House Finch, among the 84 genes identified under selection, 13 were clustered in the putative inversion in the middle region of chromosome 3 (Table S10). Within the top-10 significant GO terms (Table S9) we find terms related with development processes, morphogenesis and regulation of biochemical pathways.

**Table 2:** Number of genes detected in the outlier regions detected with *F*_st_ and XP-EHH scans (and *d*_*xy*_ for the Red-billed Chough) and their overlap. Number of genes available and feasible for the GO enrichment analysis and the number of outlier genes that were included in feasible genes (see Table S10 for genes IDs).

Species	F_st_ / *d*_*xy*_ Genes	XP-EHH Genes	Overlap	Available Genes	Feasible for GO	Feasible outliers
Red-billed Chough	2 / 96	8	0	20,580	8,788	4
Common Chaffinch	63	8	1	16,563	9,242	48
Dark-eyed Junco	15	0	0	17,038	9,410	9
House Finch	84	0	0	16,563	9,242	46

## Discussion

Our comparative analysis of mainland and insular populations of four passerine species yielded shared patterns of demographic history and divergence of morphological traits consistent with the island rule, in contrast to species-specific patterns of genome-wide variation in the studied genomic variables. Relative to the mainland, all insular populations showed changes in body size, and suffered reductions in effective population size and genetic diversity, patterns that are consistent with previous findings [24, 62, 71]. While our focus was on body size, given its well-established association to island colonization, it’s important to note that there are other traits, such as plumage color or vocalizations, that showed noticeable differences between populations [76, 78]. The process of insular colonization is usually initiated by a small group of individuals, and the resulting genetic drift, combined with the small size of the island’s geographic area, leads to a small effective population size and low genetic diversity [24, 128]. Among the four species, the Red-billed Chough showed the smallest effective population size in both insular and mainland populations, which corresponds to the lowest levels of genetic diversity. In the mainland, this species has shown marked levels of genetic structure in the absence of geographic barriers, suggesting that social barriers due to complex behavioral interactions may constrain gene flow and thus the effective size of local populations [129]; the insular population is unlikely to be an exception [77].

While our focus was on body size—given its well-established connection to island colonization—it’s important to recognize that body size is a composite of multiple traits, each contributing differently across species, as observed in the varying PCA loadings. Additionally, other phenotypes, such as plumage divergence, also showed noticeable differences between populations. However, due to the complexity of comparing these traits across species, our primary investigation centered on body size, in line with previous findings of reduced effective population size and genetic diversity in insular populations [24, 60, 69].

### Phenotypic divergence upon island colonization

Using PC1 and mean differences in tarsus length, as proxies for structural body size in birds [130–133], we found that the three smaller passerines increased in size and the larger species suffered a size reduction upon island colonization. This is consistent with the island rule, which posits that small birds evolve towards a larger size and large birds towards a smaller size upon island colonization [62, 70]. However, the difference in the House Finch tarsus length among insular and mainland populations was not significant probably due to the small sample size. Regarding beak size, we find that insular individuals from the small sized and short-billed species show longer bills than their mainland counterparts whereas the insular population of the long-billed chough species shows a reduction in bill length. All the species show also differences in at least other bill dimension; however, the Red-billed Chough is the only one in which the change is in the opposite direction, showing shorter but wider bills on the island. The beak is both a feeding and thermoregulatory structure with great evolutionary potential that allows birds to quickly adapt to new environmental conditions [134] and therefore plays a fundamental role in avian fitness [135–140].

### Differing patterns of genomic divergence

Finding shared patterns of genomic variation and common regions of differentiation at the intra- or inter-specific levels has been of major interest to understand the mechanisms underlying divergence [38, 43, 44]. These shared divergent regions across taxa are particularly interesting when differentiation evolved independently in unrelated lineages [47]. Our comparative analysis of island-mainland populations in four passerine species showed a lack of parallelism in their respective genomic landscapes. In all four species, regions of higher divergence and genetic diversity are located in the microchromosomes, which have relatively higher recombination rates and higher gene content [141]. We also found highly differentiated genomic regions in all four species that were often associated with reduced genetic diversity, which could be the result of drift or/and selection either in the ancestral or the current populations. The lack of congruence in the location of these regions along the genome could indicate that drift likely played a role in the divergence, that different traits are under selection, or that due to the polygenic nature of traits the four species adapted to their respective insular environments in different ways, through genetic changes at different loci. Moreover, patterns of recombination rate in these regions suggest that the genomic mechanisms generating these patterns, which include chromosomal inversions, and historical factors like recurrent selection, differ in each of the four species.

### Demographic history and functional genomic analysis

According to our demographic analysis, the divergence between Red-billed Choughs on La Palma and the Iberian Peninsula took place around 30,000 years ago, considering a generation time of two years. A previous study [77] estimated the divergence event in a similar time range, within the last 10,000 years using mitochondrial data and around 30,000 years using iMa2, however they used a generation time of 6 years based on mainland data. If we apply that value, the divergence time estimate changes to around 110,000 years. The Red-billed Chough also shows the smallest effective population size and lowest genetic diversity. This reduced genetic diversity also results in an inflated relative divergence [25, 142], causing a high baseline to detect outliers while the absolute divergence remains low. The scan for selective sweeps, which is more efficient in detecting recent divergence, revealed clear peaks along the genome, yet those peaks are near regions with no coverage and therefore we are considering them to be artifacts. The recent divergence of the Red-billed Chough is evident due to the low divergence across the genome, with a mean *d*_*xy*_ value of 0.002. However, this value decreases drastically to 9·10⁻⁴ when the highly divergent region on chromosome 17 is excluded. We hypothesize that this region could represent a neo-sex chromosome because of preliminary sex-related differences found (unpublished data), though further research is required to confirm this. Neo-sex chromosomes have been observed in several bird species within the Sylvioidea superfamily, often resulting from fusions or translocations of autosomes and sex chromosomes [143]. Similarly, a neo-sex chromosome was recently discovered in the genus *Zosterops*, formed by the fusion of the W chromosome and chromosome 4 A [144]. Confirmation of a neo-sex chromosome in the Red-billed Chough would be important because, to date, no autosome-sex chromosome fusions have been observed within the Corvoidea superfamily. In this study, only males were included, which limits our ability to test this hypothesis further. Among the top ten GO terms of the genes within the absolute divergence peak we find two related with ubiquitination which has been found to be an important signaling mechanism controlling several physiological and pathological processes [145].

The Common Chaffinch of La Palma was found to have diverged from its mainland relatives around 0.8–0.9 my ago, which is in agreement with previous reconstructions of the species evolutionary history [78]. A study of the entire Common Chaffinch radiation across the Atlantic archipelagos revealed that it first colonized Azores, then Madeira and finally the Canary Islands [78]. This sequential colonization of isolated archipelagos has left a genomic signature of recurrent selection along the genome, leading to regions with low absolute divergence due to selection in the ancestor, that were subsequently selected in the daughter populations, reducing genetic diversity and generating *F*_st_ peaks [40]. This recurrent-selection model fits well with the known colonization history, as the first selective episode probably occurred upon colonization of the Azores, and then at every subsequent colonization step between islands, where successive selective events at the same genomic regions likely led to a loss of genetic diversity. Among the genes associated with outlier loci there were several involved in metabolism (i.e., *kars1*, *nfrkb*), four involved in pigmentation (ap3b1, hps6, ric1 and atrn) [146], and three related to singing (*chrm5*, *mrps27*, *ube2d3*) [147–151]. Within the top-ten significant GO terms we detected “positive regulation of endosome organization” and endosomes play an important role in neural development [151]. We also find the term “regulation of protein localization to adherens junction” and it has been shown that cell adhesion plays an important role in tissue morphogenesis [152].

In the Dark-eyed Junco, the demographic inference revealed that the insular population on Guadalupe diverged around 400,000 years ago, which is similar to previous estimates [76]. The differentiated regions were mainly distributed at the ends of chromosomes, coinciding with telocentric centromeres, as previously found in Swainson’s thrushes [153]. Consistent with this pattern, among the top-ten GO terms we identified several that were related to the centrosomes, increasingly recognized as signaling machines capable of regulating many cellular functions [154].

In the House Finch, the genomic landscape showed signatures of different processes. Despite the recent divergence time between mainland and Guadalupe Island populations, estimated at about 100,000 years before present, we did no detect signatures of significant selective sweeps. The large region showing high differentiation and very low recombination in chromosome 3 likely represents a major chromosomal inversion. Upon colonization of a new environment, such as an island, a chromosomal inversion can rapidly become fixed or prevalent within the population, especially if it provides an adaptive advantage [155]. If there is subsequent gene flow between island and mainland populations, the inversion can act as a barrier to genetic exchange by reducing recombination. This suppression of recombination facilitates the maintenance of distinct genomic regions that might be beneficial in the local environment, thus reinforcing divergence between the island and mainland populations [156]. Genomic islands of differentiation could be generated by chromosomal rearrangements that cluster highly differentiated loci together due to genomic hitchhiking [114, 157]. However, that could represent either a group of adaptive alleles or several neutral loci linked to a focal selected allele [157]. Several studies have found regions highly diverged within chromosomal inversions [114, 158–160]. In this case, 13 genes putatively under selection were found within the inversion. One of those genes (Fig. 4) is related to facial morphology and related disorders [161] and the *gtf3c6* gene was found to be a candidate involved in sexual selection [162]. Another interesting candidate is the *Iyd* gene, which is also found within an inversion in chromosome 2 in the White-throated Sparrow (*Zonotrichia albicollis*) and has shown differences in expression between two morphs that differed in territorial aggression including song [163]. Within the top-ten significant GO terms, we found “growth plate cartilage chondrocyte morphogenesis”, which is involved in skeletal development and morphogenesis and regulated by multiple signaling pathways, including the bone morphogenetic proteins (BMP; [160, 164]) and Wingless/int.1 molecules (Wnt; [165]) that are known to be involved in facial development in different organisms including beak morphology in birds [166, 167].

Here we studied four cases of island-mainland divergence in passerine species that have colonized oceanic islands and share morphological modifications likely caused by similar selective pressures, and asked whether the genetic landscapes between species were also similar. Our general result in this respect is that the regions of the genome showing evidence of divergence under directional selection are lineage specific, suggesting that the genetic divergence is different in each case, so that evidence for repeated evolution at the genomic level appears to be lacking [43]. Even if the same regions had been detected as putatively under selection or with shared genomic features involved in genomic differentiation, such as the stable recombination landscape in avian lineages [50], it would be difficult to determine whether that pattern is generated by directional selection or by background and linked selection. Despite examples showing that few loci of large effect can drive adaptive divergence in complex traits, such as the bill (e.g., [168]), selection is likely to act on many loci of small effect due to the polygenic nature of most adaptive traits [169, 170]. Consequently, convergent phenotypes could in fact be due to divergent genotypes. Several examples to date show that phenotypic change in a given trait can be driven by different sets of genes, such as mouth morphology in cichlid fishes [171], or color pattern in mice [172, 173] and flies [174]. Even though the outlier genes differ among species, there could be common significant GO terms because different genes share functions and pathways. Interestingly, between the Common Chaffinch and the House Finch we found similar GO terms related to tRNA aminoacylation, and between the Red-billed Chough and the Common Chaffinch we also found the common term “protein autoubiquitination”. Remarkably, we found that in all four species, GO terms are mostly related to gene regulation, such as protein ubiquitination, transmembrane transport, and regulation of cellular localization, which are crucial for maintaining various physiological and developmental processes. Recently, Monroe et al. [175] reported that mutations occur less often in functional regions of the genome, and that epigenomic and physical chromosomal features account for the position of the mutations. In our case, most of the terms related to outlier loci are involved in regulatory and signaling pathways, suggesting that changes in gene regulation, instead of specific core genes, may be the main drivers of divergence. Currently, several models are being developed to understand the role of gene regulation in the evolution of complex traits [19, 176], implying that regulatory regions are disproportionately targeted by polygenic selection, highlighting the key role of gene regulatory networks in evolution [177].

## Electronic supplementary material

Below is the link to the electronic supplementary material.

Supplementary Material 1

## Data Availability

Resequencing raw data is deposited at NCBI under the SRA data projects PRJNA661201 (for common chaffinch mainland population) with accession numbers SAMN16094451-SAMN16094459 and PRJNA1077913 with accession numbers SAMN39984864- SAMN39984947, for the common chaffinch insular population and both populations from the rest of the species, see Table S1 for details) and the datasets, are deposited in Figshare (https://doi.org/10.6084/m9.figshare.21590673).
