# Supplementary material for: Repeated evolution on oceanic islands: comparative genomics reveals species-specific processes in birds
Source: BMC Ecol Evol. 2024 Nov 8;24:140. doi: 10.1186/s12862-024-02320-4 (PMC11545622; doi:10.1186/s12862-024-02320-4)
Supplement: Supplementary file 1 — Supplementary Material 1 [file 12862_2024_2320_MOESM1_ESM.docx]

## Supplementary Materials

## Tables

Table S1. Species, individual IDs, sampling population and accession numbers for the four species from mainland and insular populations: red-billed chough, common chaffinch, dark-eyed junco and house finch.

| **Species** | **WGS ID** | **Individuals** | **Mainland/Island** | **Population** | **Accession number** |
| --- | --- | --- | --- | --- | --- |
| Red-billed chough | MOCHO1 | 2ST | Mainland | Los Monegros, Iberia | SAMN39984864 |
| Red-billed chough | MOCHO2 | 25W | Mainland | Los Monegros, Iberia | SAMN39984865 |
| Red-billed chough | MOCHO3 | 14R | Mainland | Los Monegros, Iberia | SAMN39984866 |
| Red-billed chough | MOCHO4 | 26X | Mainland | Los Monegros, Iberia | SAMN39984867 |
| Red-billed chough | MOCHO5 | 28R | Mainland | Los Monegros, Iberia | SAMN39984868 |
| Red-billed chough | MOCHO6 | 26C | Mainland | Los Monegros, Iberia | SAMN39984869 |
| Red-billed chough | MOCHO7 | 26H | Mainland | Los Monegros, Iberia | SAMN39984870 |
| Red-billed chough | MOCHO8 | 376 | Mainland | Los Monegros, Iberia | SAMN39984871 |
| Red-billed chough | MOCHO9 | 28U | Mainland | Los Monegros, Iberia | SAMN39984872 |
| Red-billed chough | MOCHO10 | 28T | Mainland | Los Monegros, Iberia | SAMN39984873 |
| Red-billed chough | MOCHO11 | 26A | Mainland | Los Monegros, Iberia | SAMN39984874 |
| Red-billed chough | MOCHO12 | 182 | Mainland | Los Monegros, Iberia | SAMN39984875 |
| Red-billed chough | PACHO1 | 312 | Island | La Palma, Canary Islands | SAMN39984876 |
| Red-billed chough | PACHO2 | 5J8 | Island | La Palma, Canary Islands | SAMN39984877 |
| Red-billed chough | PACHO3 | 4C3 | Island | La Palma, Canary Islands | SAMN39984878 |
| Red-billed chough | PACHO4 | 4XH | Island | La Palma, Canary Islands | SAMN39984879 |
| Red-billed chough | PACHO5 | 5J3 | Island | La Palma, Canary Islands | SAMN39984880 |
| Red-billed chough | PACHO6 | 311 | Island | La Palma, Canary Islands | SAMN39984881 |
| Red-billed chough | PACHO7 | 4XX | Island | La Palma, Canary Islands | SAMN39984882 |
| Red-billed chough | PACHO8 | 4X6 | Island | La Palma, Canary Islands | SAMN39984883 |
| Red-billed chough | PACHO9 | 5J5 | Island | La Palma, Canary Islands | SAMN39984884 |
| Red-billed chough | PACHO10 | 38A | Island | La Palma, Canary Islands | SAMN39984885 |
| Red-billed chough | PACHO11 | 4CA | Island | La Palma, Canary Islands | SAMN39984886 |
| Red-billed chough | PACHO12 | 4X2 | Island | La Palma, Canary Islands | SAMN39984887 |
| Common chaffinch | CHASE1 | 16-130 | Mainland | Segovia, Iberia | SAMN16094451 |
| Common chaffinch | CHASE2 | 16-135 | Mainland | Segovia, Iberia | SAMN16094452 |
| Common chaffinch | CHASE3 | 16-100 | Mainland | Segovia, Iberia | SAMN16094453 |
| Common chaffinch | CHASE4 | 18-812 | Mainland | Segovia, Iberia | SAMN16094454 |
| Common chaffinch | CHASE5 | 18-813 | Mainland | Segovia, Iberia | SAMN16094455 |
| Common chaffinch | CHASE6 | 18-814 | Mainland | Segovia, Iberia | SAMN16094456 |
| Common chaffinch | CHASE7 | 18-815 | Mainland | Segovia, Iberia | SAMN16094457 |
| Common chaffinch | CHASE8 | 18-816 | Mainland | Segovia, Iberia | SAMN16094458 |
| Common chaffinch | CHASE9 | 18-826 | Mainland | Segovia, Iberia | SAMN16094459 |
| Common chaffinch | CHATI1 | 16-003 | Island | La Palma, Canary Islands | SAMN39984888 |
| Common chaffinch | CHATI2 | 16-006 | Island | La Palma, Canary Islands | SAMN39984889 |
| Common chaffinch | CHATI3 | 16-007 | Island | La Palma, Canary Islands | SAMN39984890 |
| Common chaffinch | CHATI4 | 16-008 | Island | La Palma, Canary Islands | SAMN39984891 |
| Common chaffinch | CHATI5 | 16-009 | Island | La Palma, Canary Islands | SAMN39984892 |
| Common chaffinch | CHATI6 | 16-011 | Island | La Palma, Canary Islands | SAMN39984893 |
| Common chaffinch | CHATI7 | 16-012 | Island | La Palma, Canary Islands | SAMN39984894 |
| Common chaffinch | CHATI8 | 16-013 | Island | La Palma, Canary Islands | SAMN39984895 |
| Common chaffinch | CHATI9 | 16-015 | Island | La Palma, Canary Islands | SAMN39984896 |
| Common chaffinch | CHATI10 | 16-016 | Island | La Palma, Canary Islands | SAMN39984897 |
| Common chaffinch | CHATI11 | 16-019 | Island | La Palma, Canary Islands | SAMN39984898 |
| Common chaffinch | CHATI12 | 16-021 | Island | La Palma, Canary Islands | SAMN39984899 |
| Dark-eyed junco | ORJU1 | 14-0529 | Mainland | California | SAMN39984900 |
| Dark-eyed junco | ORJU2 | 14-0530 | Mainland | California | SAMN39984901 |
| Dark-eyed junco | ORJU3 | 14-0531 | Mainland | California | SAMN39984902 |
| Dark-eyed junco | ORJU4 | 14-0532 | Mainland | California | SAMN39984903 |
| Dark-eyed junco | ORJU5 | 14-0533 | Mainland | California | SAMN39984904 |
| Dark-eyed junco | ORJU6 | 14-0534 | Mainland | California | SAMN39984905 |
| Dark-eyed junco | ORJU7 | 14-0535 | Mainland | California | SAMN39984906 |
| Dark-eyed junco | ORJU8 | 14-0536 | Mainland | California | SAMN39984907 |
| Dark-eyed junco | ORJU9 | 14-0539 | Mainland | California | SAMN39984908 |
| Dark-eyed junco | ORJU10 | 14-0542 | Mainland | California | SAMN39984909 |
| Dark-eyed junco | ORJU11 | 14-0543 | Mainland | California | SAMN39984910 |
| Dark-eyed junco | ORJU12 | 14-0544 | Mainland | California | SAMN39984911 |
| Dark-eyed junco | GUJU1 | 17-218 | Island | Guadalupe | SAMN39984912 |
| Dark-eyed junco | GUJU2 | 17-339 | Island | Guadalupe | SAMN39984913 |
| Dark-eyed junco | GUJU3 | 17-358 | Island | Guadalupe | SAMN39984914 |
| Dark-eyed junco | GUJU4 | 17-367 | Island | Guadalupe | SAMN39984915 |
| Dark-eyed junco | GUJU5 | 17-396 | Island | Guadalupe | SAMN39984916 |
| Dark-eyed junco | GUJU6 | 17-397 | Island | Guadalupe | SAMN39984917 |
| Dark-eyed junco | GUJU7 | 17-398 | Island | Guadalupe | SAMN39984918 |
| Dark-eyed junco | GUJU8 | 17-342 | Island | Guadalupe | SAMN39984919 |
| Dark-eyed junco | GUJU9 | 17-343 | Island | Guadalupe | SAMN39984920 |
| Dark-eyed junco | GUJU10 | 17-267 | Island | Guadalupe | SAMN39984921 |
| Dark-eyed junco | GUJU11 | 17-275 | Island | Guadalupe | SAMN39984922 |
| Dark-eyed junco | GUJU12 | 17-225 | Island | Guadalupe | SAMN39984923 |
| House finch | CAHO1 | DX-2 | Mainland | California | SAMN39984924 |
| House finch | CAHO2 | DV-34 | Mainland | California | SAMN39984925 |
| House finch | CAHO3 | W-22 | Mainland | California | SAMN39984926 |
| House finch | CAHO4 | DV-5 | Mainland | California | SAMN39984927 |
| House finch | CAHO5 | W-10 | Mainland | California | SAMN39984928 |
| House finch | CAHO6 | DX-42 | Mainland | California | SAMN39984929 |
| House finch | CAHO7 | DX-29 | Mainland | California | SAMN39984930 |
| House finch | CAHO8 | W-18 | Mainland | California | SAMN39984931 |
| House finch | CAHO9 | 13-022 | Mainland | Sierra Juarez | SAMN39984932 |
| House finch | CAHO10 | 13-023 | Mainland | Sierra Juarez | SAMN39984933 |
| House finch | CAHO11 | W-29 | Mainland | California | SAMN39984934 |
| House finch | CAHO12 | DX-45 | Mainland | California | SAMN39984935 |
| House finch | GUHO1 | 17-228 | Island | Guadalupe | SAMN39984936 |
| House finch | GUHO2 | 17-240 | Island | Guadalupe | SAMN39984937 |
| House finch | GUHO3 | 17-246 | Island | Guadalupe | SAMN39984938 |
| House finch | GUHO4 | 17-254 | Island | Guadalupe | SAMN39984939 |
| House finch | GUHO5 | 17-274 | Island | Guadalupe | SAMN39984940 |
| House finch | GUHO6 | 17-341 | Island | Guadalupe | SAMN39984941 |
| House finch | GUHO7 | 17-344 | Island | Guadalupe | SAMN39984942 |
| House finch | GUHO8 | 17-238 | Island | Guadalupe | SAMN39984943 |
| House finch | GUHO9 | 17-419 | Island | Guadalupe | SAMN39984944 |
| House finch | GUHO10 | 17-374 | Island | Guadalupe | SAMN39984945 |
| House finch | GUHO11 | 17-235 | Island | Guadalupe | SAMN39984946 |
| House finch | GUHO12 | 11-0126 | Island | Guadalupe | SAMN39984947 |

Table S2. Correspondence between the chromosomes of the Zebra finch (*Taeniopygia guttata*) genome assembly (bTaeGut2.pat.W.v2), including the chromosome name and Refseq ID and the scaffolds of the reference genomes for the common chaffinch (*Fringilla coelebs*), the junco (*Junco hyemalis*) and the New Caledonian crow (*Corvus Moneduloides*).

| Chrom. | RefSeq Zebra finch | Common chaffinch assembly | Junco assembly | New Caledonian crow assembly |
| --- | --- | --- | --- | --- |
| 1 | NC_044998.1 | ScDMRwT_581_HRSCAF_676 | ScoVZU6_963__HRSCAF___984 | NC_045477.1 |
| 1A | NC_044999.1 | ScDMRwT_3155_HRSCAF_3635 | ScoVZU6_1238__HRSCAF___1266 | NC_045479.1 |
| 2 | NC_045000.1 | ScDMRwT_473_HRSCAF_551 | ScoVZU6_2668__HRSCAF___2714 | NC_045476.1 |
| 3 | NC_045001.1 | ScDMRwT_2155_HRSCAF_2491 | ScoVZU6_1491__HRSCAF___1522 | NC_045478.1 |
| 4 | NC_045002.1 | ScDMRwT_1738_HRSCAF_2009 | ScoVZU6_2655__HRSCAF___2701 | NC_045480.1 |
| 5 | NC_045004.1 | ScDMRwT_3240_HRSCAF_3767 | ScoVZU6_3544__HRSCAF___3601 | NC_045489.1 |
| 4A | NC_045003.1 | ScDMRwT_1904_HRSCAF_2197 | ScoVZU6_1225__HRSCAF___1252 | NC_045481.1 |
| 6 | NC_045005.1 | ScDMRwT_2188_HRSCAF_2528 | ScoVZU6_982__HRSCAF___1003 | NC_045483.1 |
| 7 | NC_045006.1 | ScDMRwT_1364_HRSCAF_1572 | ScoVZU6_3251__HRSCAF___3306 | NC_045482.1 |
| 8 | NC_045007.1 | ScDMRwT_804_HRSCAF_937 | ScoVZU6_2192__HRSCAF___2234 | NC_045484.1 |
| 9 | NC_045008.1 | ScDMRwT_200_HRSCAF_244 | ScoVZU6_201__HRSCAF___207 | NC_045485.1 |
| 10 | NC_045009.1 | ScDMRwT_1361_HRSCAF_1569 | ScoVZU6_4447__HRSCAF___4518 | NC_045488.1 |
| 11 | NC_045010.1 | ScDMRwT_1149_HRSCAF_1324 | ScoVZU6_3582__HRSCAF___3639 | NC_045487.1 |
| 12 | NC_045011.1 | ScDMRwT_2095_HRSCAF_2420 | ScoVZU6_4448__HRSCAF___4521 | NC_045486.1 |
| 13 | NC_045012.1 | ScDMRwT_1072_HRSCAF_1239 | ScoVZU6_2057__HRSCAF___2097 | NC_045490.1 |
| 14 | NC_045013.1 | ScDMRwT_2434_HRSCAF_2805 | ScoVZU6_3803__HRSCAF___3864 | NC_045491.1 |
| 15 | NC_045014.1 | ScDMRwT_1873_HRSCAF_2164 | ScoVZU6_4455__HRSCAF___4539 | NC_045493.1 |
| 17 | NC_045015.1 | ScDMRwT_2070_HRSCAF_2390 | ScoVZU6_1929__HRSCAF___1968 | NC_045496.1 |
| 18 | NC_045016.1 | ScDMRwT_1489_HRSCAF_1712 | ScoVZU6_582__HRSCAF___593 | NC_045494.1 |
| 19 | NC_045017.1 | ScDMRwT_2017_HRSCAF_2332 | ScoVZU6_2973__HRSCAF___3026 | NC_045495.1 |
| 20 | NC_045018.1 | ScDMRwT_211_HRSCAF_256 | ScoVZU6_1043__HRSCAF___1065 | NC_045492.1 |
| 21 | NC_045019.1 | ScDMRwT_1489_HRSCAF_1712 | ScoVZU6_3047__HRSCAF___3100 | NC_045497.1 |
| 22 | NC_045020.1 | ScDMRwT_1489_HRSCAF_1712 | ScoVZU6_1118__HRSCAF___1143 | NC_045502.1 |
| 23 | NC_045021.1 | ScDMRwT_1489_HRSCAF_1712 | ScoVZU6_300__HRSCAF___308 | NC_045498.1 |
| 24 | NC_045022.1 | ScDMRwT_1489_HRSCAF_1712 | ScoVZU6_957__HRSCAF___978 | NC_045500.1 |
| 25 | NC_045023.1 | ScDMRwT_1489_HRSCAF_1712 | ScoVZU6_1653__HRSCAF___1687 | NC_045504.1 |
| 26 | NC_045024.1 | ScDMRwT_1489_HRSCAF_1712 | ScoVZU6_3291__HRSCAF___3346 | NC_045499.1 |
| 27 | NC_045025.1 | ScDMRwT_1489_HRSCAF_1712 | ScoVZU6_3522__HRSCAF___3579 | NC_045501.1 |
| 28 | NC_045026.1 | ScDMRwT_1489_HRSCAF_1712 | ScoVZU6_4457__HRSCAF___4541 | NC_045503.1 |
| Z | NC_045027.1 | ScGsoED_1_HRSCAF_2 | ScU1KuS_2__HRSCAF___21 | NC_045511.1 |

Table S3. Mean values (x̄), standard deviation (sd) and ANOVA test including *F* statistic, degrees of freedom (df) and *p*-value for the red-billed chough, common chaffinch, junco and house finch morphological traits and PC1 including all variables from the insular and mainland populations. The sample size for each population is specified for each species and treatment (n). The compared traits are wing length, tail length, tarsus length, culmen, bill length, depth and width. Statistically significant *p*-values are in bold.

|  | **Red-Billed Chough** | | | | **Common chaffinch** | | | |
| --- | --- | --- | --- | --- | --- | --- | --- | --- |
| **Trait** | **x̄_island_ ± sd** | **x̄_continent_ ± sd** | ***F* value _df_** | ***p*-value** | **x̄_island_ ± sd** | **x̄_continent_ ± sd** | ***F* value _df_** | ***p*-value** |
|  | **n=25** | **n=25** |  |  | **n=25** | **n=25** |  |  |
| Wing length | 287.04 ± 8.18 | 296.24 ± 7.19 | 17.85 _1,48_ | **<.0001** | 86.28 ± 1.52 | 85.94 ± 1.79 | 0.52 _1,48_ | 0.47 |
| Tail length | 138.76 ± 5.74 | 143.6 ± 5.43 | 9.38 _1,48_ | **<.01** | 75.05 ± 1.61 | 66.77 ± 2.72 | 171.3 _1,48_ | **<.0001** |
| Tarsus length | 52.96 ± 1.66 (n=24) | 56.42 ± 1.26 | 67.84 _1,47_ | **<.0001** | 21.8 ± 0.54 | 18.26 ± 0.52 | 559 _1,48_ | **<.0001** |
| Culmen | 59.38 ± 2.54 (n=24) | 61.75 ± 2.85 | 9.37 _1,47_ | **<.01** | 11.78 ± 0.44 | 9.76 ± 0.35 | 322.7 _1,48_ | **<.0001** |
| Bill length | - | - | - | - | 15.47 ± 0.61 | 12.53 ± 0.41 | 403.4 _1,48_ | **<.0001** |
| Bill depth | 13.29 ± 0.49 (n=24) | 13.32 ± 0.49 | 0.03 _1,47_ | 0.86 | 8.09 ± 0.26 | 6.73 ± 0.21 | 417.9 _1,48_ | **<.0001** |
| Bill width | 13.70 ± 0.66 (n=23) | 12.42 ± 0.69 | 42.90 _1,46_ | **<.0001** | 6.24 ± 0.17 | 5.39 ± 0.22 | 242.6 _1,48_ | **<.0001** |
| PC1 | 1.18 ± 1.12 | -1.04 ± 1.01 | 50.7 _1,45_ | **<.0001** | -2.26 ± 0.47 | 2.26 ± 0.47 | 923.3 _1,48_ | **<.0001** |
|  | **Junco sp.** | | | | **House finch** | | | |
| **Trait** | **x̄_island_ ± sd** | **x̄_continent_ ± sd** | ***F* value _df_** | ***p*-value** | **x̄_island_ ± sd** | **x̄_continent_ ± sd** | ***F* value _df_** | ***p*-value** |
|  | **n=12** | **n=12** |  |  | **n=8** | **n=5** |  |  |
| Wing length | 68.59 ± 1.31 | 71.38 ± 1.76 | 19.27 _1,22_ | **<.0005** | 81.13 ± 1.57 | 79.4 ± 2.38 | 2.51 _1,11_ | 0.14 |
| Tail length | 62.01 ± 2.06 | 62.77 ± 2.93 | 0.53 _1,22_ | 0.47 | 65.15 ± 1.65(n=7) | 62.86 ± 4.55 | 1.56 _1,10_ | 0.24 |
| Tarsus length | 21 ± 0.41 | 20.11 ± 0.41 | 27.56 _1,22_ | **<.0001** | 19.29 ± 0.51 | 17.5 ± 0.46 | 41.14 _1,11_ | **<.0001** |
| Culmen | 9.8 ± 0.42 | 8.68 ± 0.42 | 43.13 _1,22_ | **<.0001** | 9.95 ± 0.33 | 8.3 ± 0.62 | 40.06 _1,11_ | **<.0001** |
| Bill length | 13.08 ± 0.46 | 11.52 ± 0.44 | 72.1 _1,22_ | **<.0001** | 12.54 ± 0.37 | 10.88 ± 0.13 | 90.56 _1,11_ | **<.0001** |
| Bill depth | 6.14 ± 0.16 | 5.69 ± 0.28 | 23.08 _1,22_ | **<.0001** | 10 ± 0.35 | 7.88 ± 0.20 | 145.2 _1,11_ | **<.0001** |
| Bill width | 4.43 ± 0.15 | 4.56 ± 0.22 | 2.66 _1,22_ | 0.12 | 7.71 ± 0.24 | 6.44 ± 0.19 | 97.74 _1,11_ | **<.0001** |
| PC1 | 1.72 ± 0.76 | -1.72 ± 0.63 | 145.2 _1,22_ | **<.0001** | -2.44 ± 0.20 | 1.22 ± 1.63 | 19.14 _1,10_ | **<.01** |
|  |  |  |  |  |  |  |  |  |

**Table S4.** Number of sites obtained in the variant calling for each species performed with BCFTOOLS, including the total number of sites (variant and invariant), only the variant sites and the total number of sites and variant sites after filtering.

| **Species** | **Total sites** | **Variant sites** | **Filtered dataset** | **Filtered variants** |
| --- | --- | --- | --- | --- |
| Red-billed chough | 1,026,780,642 | 13,411,090 | 977,729,328 | 2,618,783 |
| Common chaffinch | 994,789,329 | 52,711,626 | 965,104,289 | 25,441,259 |
| Dark-eyed junco | 1,001,453,032 | 42,444,447 | 972,975,172 | 16,520,306 |
| House finch | 973,127,256 | 43,257,766 | 944,413,769 | 19,649,701 |

Table S5. Detection of chromosomal inversions. Outlier multidimensional scaling (MDS) values detected by *lostruct* using 1,000bp windows for the dark-eyed junco, the common chaffinch and the house finch, considered as putative inversions.

| MDS | Chr | Scaffold | Start | End | Outlier  windows | PC1  Variance  (%) | PC2  Variance  (%) | Proportion of  between clusters  sum of squares |
| --- | --- | --- | --- | --- | --- | --- | --- | --- |
| **Common Chaffinch** | | | | | | | | |
| MDS02 | 20 | ScDMRwT_211_HRSCAF_256 | 4051397 | 11870498 | 140 | 29.68 | 5.92 | 0.9967 |
| MDS05 | 10 | ScDMRwT_1361_HRSCAF_1569 | 818326 | 15323400 | 100 | 31.26 | 6.6 | 0.9954 |
| **Dark-eyed junco** | | | | | | | | |
| MDS02 | 5 | ScoVZU6_3544__HRSCAF___3601 | 7092090 | 25039949 | 11 | 30.12 | 7.69 | 0.9814 |
| MDS25 | 5 | ScoVZU6_3544__HRSCAF___3601 | 11875088 | 46088461 | 44 | 34.15 | 14.98 | 0.9941 |
| MDS28 | 3 | ScoVZU6_1491__HRSCAF___1522 | 94713851 | 101757387 | 48 | 33.85 | 14.86 | 0.9918 |
| MDS31 | 3 | ScoVZU6_1491__HRSCAF___1522 | 91846156 | 94371078 | 42 | 33.13 | 21.07 | 0.9783 |
| MDS33 | 1 | ScoVZU6_963__HRSCAF___984 | 49236367 | 93878973 | 41 | 38.3 | 19.21 | 0.9955 |
| **House finch** | | | | | | | | |
| MDS13 | 3 | ScDMRwT_2155_HRSCAF_2491 | 32955906 | 80670074 | 304 | 24.63 | 15.78 | 0.9892 |
| MDS20 | Z | ScGsoED_1_HRSCAF_2 | 41382108 | 63213746 | 21 | 34.73 | 13.13 | 0.981 |
| MDS30 | 5 | ScDMRwT_3240_HRSCAF_3767 | 18585012 | 24736215 | 4 | 43.78 | 21.62 | 0.9451 |
| MDS36 | 4 | ScDMRwT_1738_HRSCAF_2009 | 42772732 | 43082901 | 7 | 38.48 | 24.38 | 0.9603 |

Table S6. Top table: top ten GO terms associated to 36 out of the 96 outlier genes detected by *d_xy_* in the island-mainland red-billed chough comparison. Bottom table: top ten GO terms associated to 4 out of the 8 outlier genes detected by XP-EHH in the island-mainland red-billed chough comparison.The analysis was performed using the topGO package with the weight01 algorithm and the fisher test under the ontology biological process. The table includes the GO term Accession (GO.ID), the term name, the number of annotated genes (Annot.), the number of significant genes (Sign.), the number of expected genes (Exp.) and the *p*-value of the Fisher test using the weight01 algorithm.

| **GO.ID** | **Term** | **Annot.** | **Sign.** | **Exp.** | **Fisher** |
| --- | --- | --- | --- | --- | --- |
| GO:1903265 | positive regulation of tumor necrosis factor-mediated signaling pathway | 4 | 2 | 0.02 | 9.70E-05 |
| GO:0051865 | protein autoubiquitination | 51 | 3 | 0.21 | 0.0011 |
| GO:0015679 | plasma membrane copper ion transport | 1 | 1 | 0 | 0.0041 |
| GO:1904777 | negative regulation of protein localization to cell cortex | 1 | 1 | 0 | 0.0041 |
| GO:0007014 | actin ubiquitination | 1 | 1 | 0 | 0.0041 |
| GO:0090155 | negative regulation of sphingolipid biosynthetic process | 1 | 1 | 0 | 0.0041 |
| GO:1905429 | response to glycine | 1 | 1 | 0 | 0.0041 |
| GO:1902601 | silver ion transmembrane transport | 1 | 1 | 0 | 0.0041 |
| GO:1903883 | positive regulation of interleukin-17-mediated signaling pathway | 1 | 1 | 0 | 0.0041 |
| GO:1903886 | positive regulation of chemokine (C-C motif) ligand 20 production | 1 | 1 | 0 | 0.0041 |

Table S7. Top ten GO terms associated with 47 out of the 63 outlier genes detected by the *F*_st_ scan in the island-mainland common chaffinch comparison. The analysis was performed using the topGO package with the weight01 algorithm and the fisher test under the ontology biological process. The table includes the GO term Accession (GO.ID), the term name, the number of annotated genes (Annot.), the number of significant genes (Sign.), the number of expected genes (Exp.) and the *p*-value of the Fisher test using the weight01 algorithm.

| **GO.ID** | **Term** | **Annot** | **Sign** | **Exp** | **Fisher** |
| --- | --- | --- | --- | --- | --- |
| GO:1901475 | pyruvate transmembrane transport | 4 | 2 | 0.02 | 0.00013 |
| GO:0035873 | lactate transmembrane transport | 5 | 2 | 0.03 | 0.00021 |
| GO:0051865 | protein autoubiquitination | 48 | 3 | 0.25 | 0.0014 |
| GO:0090668 | endothelial cell chemotaxis to vascular endothelial growth factor | 1 | 1 | 0.01 | 0.00465 |
| GO:1904980 | positive regulation of endosome organization | 1 | 1 | 0.01 | 0.00465 |
| GO:0071765 | nuclear inner membrane organization | 1 | 1 | 0.01 | 0.00465 |
| GO:0000972 | transcription-dependent tethering of RNA polymerase II gene DNA at nuclear periphery | 1 | 1 | 0.01 | 0.00465 |
| GO:1904702 | regulation of protein localization to adherens junction | 1 | 1 | 0.01 | 0.00465 |
| GO:0006430 | lysyl-tRNA aminoacylation | 1 | 1 | 0.01 | 0.00465 |
| GO:0042307 | positive regulation of protein import into nucleus | 24 | 2 | 0.12 | 0.00547 |

Table S8. Top ten GO terms associated with 9 out of the 15 outlier genes detected by the *F*_st_ scan in the island-mainland dark-eyed junco comparison. The analysis was performed using the topGO package with the weight01 algorithm and the fisher test under the ontology biological process. The table includes the GO term Accession (GO.ID), the term name, the number of annotated genes (Annot.), the number of significant genes (Sign.), the number of expected genes (Exp.) and the *p*-value of the Fisher test using the weight01 algorithm.

| **GO.ID** | **Term** | **Annot** | **Sign** | **Exp.** | **Fisher** |
| --- | --- | --- | --- | --- | --- |
| GO:1904780 | negative regulation of protein localization to centrosome | 2 | 1 | 0 | 0.0015 |
| GO:0048692 | negative regulation of axon extension involved in regeneration | 2 | 1 | 0 | 0.0015 |
| GO:0030449 | regulation of complement activation | 2 | 1 | 0 | 0.0015 |
| GO:0061034 | olfactory bulb mitral cell layer development | 2 | 1 | 0 | 0.0015 |
| GO:0097402 | neuroblast migration | 2 | 1 | 0 | 0.0015 |
| GO:0006104 | succinyl-CoA metabolic process | 3 | 1 | 0 | 0.0022 |
| GO:0046604 | positive regulation of mitotic centrosome separation | 3 | 1 | 0 | 0.0022 |
| GO:0045869 | negative regulation of single stranded viral RNA replication via double stranded DNA intermediate | 3 | 1 | 0 | 0.0022 |
| GO:0051968 | positive regulation of synaptic transmission, glutamatergic | 3 | 1 | 0 | 0.0022 |
| GO:0090309 | positive regulation of DNA methylation-dependent heterochromatin formation | 3 | 1 | 0 | 0.0022 |

Table S9. Top ten GO terms associated with 46 out of the 84 outlier genes detected by the *F*_st_ scan in the island-mainland house finch comparison. The analysis was performed using the topGO package with the weight01 algorithm and the fisher test under the ontology biological process. The table includes the GO term Accession (GO.ID), the term name, the number of annotated genes (Annot.), the number of significant genes (Sign.), the number of expected genes (Exp.) and the *p*-value of the Fisher test using the weight01 algorithm.

| **GO.ID** | **Term** | **Annot** | **Sign** | **Exp** | **Fisher** |
| --- | --- | --- | --- | --- | --- |
| GO:0046856 | phosphatidylinositol dephosphorylation | 20 | 2 | 0.1 | 0.0042 |
| GO:0090258 | negative regulation of mitochondrial fission | 1 | 1 | 0 | 0.0049 |
| GO:0035879 | plasma membrane lactate transport | 1 | 1 | 0 | 0.0049 |
| GO:0003429 | growth plate cartilage chondrocyte morphogenesis | 1 | 1 | 0 | 0.0049 |
| GO:0072274 | metanephric glomerular basement membrane development | 1 | 1 | 0 | 0.0049 |
| GO:0051106 | positive regulation of DNA ligation | 1 | 1 | 0 | 0.0049 |
| GO:0051780 | behavioral response to nutrient | 1 | 1 | 0 | 0.0049 |
| GO:0006425 | glutaminyl-tRNA aminoacylation | 1 | 1 | 0 | 0.0049 |
| GO:1901525 | negative regulation of mitophagy | 2 | 1 | 0.01 | 0.0097 |
| GO:0099074 | mitochondrion to lysosome transport | 2 | 1 | 0.01 | 0.0097 |

Table S10. Putative outlier genes associated with genomic differentiation across four bird species. The table lists genes detected by the *F*_st_ and XP-EHH scans for the Red-billed Chough (*Pyrrhocorax pyrrhocorax*), Common Chaffinch (*Fringilla coelebs*), Dark-eyed Junco (*Junco hyemalis*), and House Finch (*Haemorhous mexicanus*). Additionally, for the Red-billed Chough, genes within the region of elevated *d_xy_* on chromosome 17 are specified. For the House Finch, genes within the putative inversion on chromosome 3 are also included. Genes shared between the Red-billed Chough *d_xy_* outliers and the House Finch *F*_st_ outliers are highlighted in blue. The common gene to the *F*_st_ and XP-EHH scans for the Common Chaffinch is shaded in green. The gene shared between the House Finch and Junco *F*_st_ outliers is highlighted in orange.

| **Red-billed Chough** | | | **Common Chaffinch** | | **Dark-eyed Junco** | **House Finch** | |
| --- | --- | --- | --- | --- | --- | --- | --- |
| ***d_xy_*** | ***F*_st_** | **XP-EHH** | ***F*_st_** | **XP-EHH** | ***F*_st_** | ***F*_st_** | **Inversion** |
| ASTN2 | LOC116437550 | IGF2BP3 | UBAP2 | LOC112530493 | ARL14EPL | LOC100220956 | PNISR |
| TRIM32 | LOC116437554 | MALSU1 | DCAF12 | PLAU | P4HA1 | LOC101232962 | LOC116808102 |
| PAPPA |  | FAM126A | NNT | LOC115490544 | NUDT13 | ERBIN | USP45 |
| TNC |  | ACOD1 | EMB | MTMR8 | OGDH | SREK1 | FIG4 |
| TNFSF8 |  | LOC116442768 | PIK3R1 | SYTL1 | LOC100225533 | SRSF11 | GTF3C6 |
| TNFSF15 |  | SHANK3 | AP3B1 | SELENBP1L3 | GOLGB1 | SRSF11 | KPNA5 |
| LOC116454345 |  | WAPL | MRPS27 | MLLT6 | LOC100223649 | MAST2 | FAM162B |
| LOC116454346 |  | LOC116453176 | RIC1 | SUGP2 | CALU | PSME3IP1 | MAN1A2 |
| TMEM268 |  |  | LURAP1L |  | SUSD4 | RIPOR1 | TRDNL |
| ATP6V1G1 |  |  | FOCAD |  | SYNE1 | LOC107052159 | IYD |
| WHRN |  |  | KIF27 |  | RALGPS1 | FBXL22 | EXO1 |
| AKNA |  |  | MCTP1 |  | TNR | LOC105759190 | RGS7 |
| STXBP1 |  |  | CZH9orf40 |  | UBXN2B | HPSE | LGALS8 |
| NIBAN2 |  |  | CSNK1G3 |  | LRIF1 | SLC35E4 |  |
| SLC31A2 |  |  | SHB |  | MORC2 | IQCD |  |
| LOC116454419 |  |  | APC |  |  | OSBP2 |  |
| CACNA1B |  |  | LOC115491256 |  |  | LOC115497384 |  |
| EHMT1 |  |  | LOC115491192 |  |  | SCARF2 |  |
| ARRDC1 |  |  | LOC115491069 |  |  | DGCR6L |  |
| LOC116454406 |  |  | GOLGA4 |  |  | SLC7A4 |  |
| LOC116454461 |  |  | PPP1R17 |  |  | LOC427826 |  |
| NELFB |  |  | SLC12A7 |  |  | POLE |  |
| LOC116454407 |  |  | SLC12A4 |  |  | PLA2G3 |  |
| LOC116454408 |  |  | NFX1 |  |  | INPP5J |  |
| TUBB4B |  |  | BBS9 |  |  | SMTN |  |
| FAM166A |  |  | SYNJ1 |  |  | MORC2 |  |
| LOC116454259 |  |  | GRAMD1C |  |  | BAZ1B |  |
| TOR4A |  |  | ADAT1 |  |  | LOC116809107 |  |
| ANAPC2 |  |  | KARS1 |  |  | HIP1 |  |
| SSNA1 |  |  | USP38 |  |  | TSPOAP1 |  |
| TPRN |  |  | GAB1 |  |  | CCDC183 |  |
| NSMF |  |  | UBE2D3 |  |  | SBDS |  |
| PNPLA7 |  |  | UBE2D2 |  |  | LOC116809154 |  |
| MRPL41 |  |  | ATRN |  |  | CLIC3 |  |
| DPH7 |  |  | LYPLAL1 |  |  | PAXX |  |
| ZMYND19 |  |  | EHBP1 |  |  | TMEM141 |  |
| RPL12 |  |  | ARMH3 |  |  | ENTPD2 |  |
| LOC116454552 |  |  | HPS6 |  |  | PCBP4 |  |
| LRSAM1 |  |  | LDB1 |  |  | LOC100223410 |  |
| FKBP15 |  |  | PI4K2A |  |  | LOC426023 |  |
| SLC31A1 |  |  | AVPI1 |  |  | LOC100223410 |  |
| CDC26 |  |  | TLL2 |  |  | PCBP3 |  |
| PRPF4 |  |  | LOC100228872 |  |  | GPR62 |  |
| WDR31 |  |  | CYP2C45 |  |  | PARP3 |  |
| BSPRY |  |  | PLAU |  |  | SETD5 |  |
| HDHD3 |  |  | VCL |  |  | LHFPL4 |  |
| ALAD |  |  | ACO2 |  |  | MTMR14 |  |
| POLE3 |  |  | LOC115493506 |  |  | CPNE9 |  |
| RGS3 |  |  | HSP90B1 |  |  | BRPF1 |  |
| LOC116454333 |  |  | ABCC9 |  |  | BRPF1 |  |
| LOC116454334 |  |  | PRR5 |  |  | BRD1 |  |
| TRNAT-AGU-4 |  |  | IRAG1 |  |  | LAMB2 |  |
| LOC116454506 |  |  | LYVE1 |  |  | USP19 |  |
| LOC116454507 |  |  | LOC116808484 |  |  | QARS1 |  |
| ZNF618 |  |  | CHRM5 |  |  | QRICH1 |  |
| GRIN1 |  |  | SPAG9 |  |  | IMPDH2 |  |
| LRRC26 |  |  | MBTD1 |  |  | ARIH2 |  |
| RNF224 |  |  | SLC16A3 |  |  | PNISR |  |
| CYSRT1 |  |  | SLC16A7 |  |  | PNISR |  |
| RNF208 |  |  | TARDBP |  |  | LOC116808102 |  |
| NDOR1 |  |  | PRDM10 |  |  | USP45 |  |
| TMEM203 |  |  | NFRKB |  |  | FIG4 |  |
| LOC116454210 |  |  | ST14 |  |  | GTF3C6 |  |
| LOC116454209 |  |  |  |  |  | KPNA5 |  |
| ENTPD8 |  |  |  |  |  | FAM162B |  |
| NOXA1 |  |  |  |  |  | MAN1A2 |  |
| LOC116454213 |  |  |  |  |  | TRDNL |  |
| EXD3 |  |  |  |  |  | IYD |  |
| NRARP |  |  |  |  |  | EXO1 |  |
| AMBP |  |  |  |  |  | RGS7 |  |
| RABL6 |  |  |  |  |  | LGALS8 |  |
| LOC116454473 |  |  |  |  |  | EPRS1 |  |
| AJM1 |  |  |  |  |  | MAP4K3 |  |
| PHPT1 |  |  |  |  |  | AGMAT |  |
| MAMDC4 |  |  |  |  |  | PINK1 |  |
| EDF1 |  |  |  |  |  | EPB41 |  |
| TRAF2 |  |  |  |  |  | MATN1 |  |
| FBXW5 |  |  |  |  |  | PUM1 |  |
| C8G |  |  |  |  |  | TUFT1 |  |
| LOC116454410 |  |  |  |  |  | MAGI3 |  |
| LOC116454411 |  |  |  |  |  | MAGI1 |  |
| LCNL1 |  |  |  |  |  | LRIG2 |  |
| NPDC1 |  |  |  |  |  | SLC16A1 |  |
| LOC116454474 |  |  |  |  |  |  |  |
| FUT7 |  |  |  |  |  |  |  |
| ABCA2 |  |  |  |  |  |  |  |
| CLIC3 |  |  |  |  |  |  |  |
| PAXX |  |  |  |  |  |  |  |
| TMEM141 |  |  |  |  |  |  |  |
| ENTPD2 |  |  |  |  |  |  |  |
| SAPCD2 |  |  |  |  |  |  |  |
| UAP1L1 |  |  |  |  |  |  |  |
| DPP7 |  |  |  |  |  |  |  |
| LOC116454425 |  |  |  |  |  |  |  |
| LOC116454426 |  |  |  |  |  |  |  |
| LOC116454455 |  |  |  |  |  |  |  |

## Figures

Figure S1. Synteny plots of the three reference genomes used, against the zebra finch genome (*Taeniopygia guttata*, bTaeGut2.pat.W.v2) performed with Circos (Krzywinski et al., 2009). (A) New Caledonian crow (*Corvus moneduloides*), (B) Common chaffinch (*Fringilla coelebs*) and (C) Dark-eyed junco (*Junco hyemalis*). The zebra finch is on the right hemisphere of each plot.

Figure S2. Demographic history of insular and mainland populations. The analysis was performed using Pairwise Sequentially Markovian Coalescent (PSMC). Demographic inference (dark red line) and 100 bootstrap replicates (lighter red lines) for one individual per treatment and species. The left and right columns correspond to the continent and the island individuals, respectively. The mutation rate used was of 4.6e-9 mutation/site/generation for all species, and the generation time used in all cases was two years.

Figure S3. Demographic history of insular and mainland populations. The analysis was performed using Pairwise Sequentially Markovian Coalescent (PSMC). Demographic inference (one individual per treatment and species, blue and orange lines correspond to the continent and the island individuals, respectively. The mutation rate used was of 4.6e-9 mutation/site/generation for all species, and the generation time used in all cases was one year except for the Red-billed Chough that is three years.

Figure S4. Detailed manhattan plots of the XP-EHH outlier regions for the Red-billed Chough. From top to bottom, fixation index (*F*_st_), Tajima’s D for insular and continental populations (TajD), Cross-populations Extended Haplotype Homozygosity (XP-EHH) and read depth. Chromosome numbers correspond to the Zebra finch genome (*Taeniopygia guttata*). Green dots represent outliers with the false discovery rate (FDR) set at 0.05 after applying the Benjamini and Hochberg correction, except for the XP-EHH, where the threshold is set at –log10 (*p*-value) ≥ 3. The yellow boxes highlight the XP-EHH peaks coincident with drops in Tajima’s D.

(d)

(d)

(d)

(d)

(d)

(d)

**Figure S5.** Detection of chromosomal inversions using multidimensional scaling analysis (MDS) and linkage disequilibrium (LD) heatmap. Information about the MDS outlier regions detected with lostruct for the Dark-eyed Junco, including: (a) genome plot of MDS values for the chromosomes were inversions are detected, where each dot represents a window of 1,000 SNPs and outliers are in shown in red; (b) PCA performed with SNPs in the specific region. The three clusters correspond to the two homozygote groups (blue and red) and the heterozygote group (purple); and (c) heterozygosity values for each of the groups identified in the PCA; and (d) LD heatmap of the inversion region, with a gradient from white to red indicating low to high LD, respectively.

(d)

(d)

**Figure S6.** Detection of chromosomal inversions using multidimensional scaling analysis (MDS) and linkage disequilibrium (LD) heatmap. Information about the MDS outlier regions detected with lostruct for the common chaffinch, including: (a) genome plot of MDS values for the chromosomes were inversions are detected, where each dot represents a window of 1,000 SNPs and outliers are in shown in red; (b) PCA performed with SNPs in the specific region. The three clusters correspond to the two homozygote groups (blue and red) and the heterozygote group (purple); (c) heterozygosity values for each of the groups identified in the PCA; and (d) LD heatmap of the inversion region, with a gradient from white to red indicating low to high LD, respectively.

(d)

(d)

(d)

(d)

**Figure S7.** Detection of chromosomal inversions using multidimensional scaling analysis (MDS) and linkage disequilibrium (LD) heatmap. Information about the MDS outlier regions detected with lostruct for the house finch, including: (a) genome plot of MDS values for the chromosomes were inversions are detected, where each dot represents a window of 1,000 SNPs and outliers are in shown in red; (b) PCA performed with SNPs in the specific region. The three clusters correspond to the two homozygote groups (blue and red) and the heterozygote group (purple); (c) heterozygosity values for each of the groups identified in the PCA; and (d) LD heatmap of the inversion region, with a gradient from white to red indicating low to high LD, respectively.
